# Saccharomyces cerevisiae Requires *CFF1* To Produce 4-Hydroxy-5-Methylfuran-3(2H)-One, a Mimic of the Bacterial Quorum-Sensing Autoinducer AI-2

**DOI:** 10.1128/mBio.03303-20

**Published:** 2021-03-09

**Authors:** Julie S. Valastyan, Christina M. Kraml, Istvan Pelczer, Thomas Ferrante, Bonnie L. Bassler

**Affiliations:** a Department of Molecular Biology, Princeton University, Princeton, New Jersey, USA; b Howard Hughes Medical Institute, Chevy Chase, Maryland, USA; c Lotus Separations LLC, Department of Chemistry, Princeton University, Princeton, New Jersey, USA; d Department of Chemistry, Princeton University, Princeton, New Jersey, USA; Washington University School of Medicine

**Keywords:** autoinducer, collective behavior, interspecies, quorum sensing, signaling, yeast

## Abstract

Quorum sensing is a process of cell-to-cell communication that bacteria use to orchestrate collective behaviors. Quorum sensing depends on the production, release, and detection of extracellular signal molecules called autoinducers (AIs) that accumulate with increasing cell density. While most AIs are species specific, the AI called AI-2 is produced and detected by diverse bacterial species, and it mediates interspecies communication. We recently reported that mammalian cells produce an AI-2 mimic that can be detected by bacteria through the AI-2 receptor LuxP, potentially expanding the role of the AI-2 system to interdomain communication. Here, we describe a second molecule capable of interdomain signaling through LuxP, 4-hydroxy-5-methylfuran-3(2H)-one (MHF), that is produced by the yeast Saccharomyces cerevisiae. Screening the S. cerevisiae deletion collection revealed Cff1p, a protein with no known role, to be required for MHF production. Cff1p is proposed to be an enzyme, with structural similarity to sugar isomerases and epimerases, and substitution at the putative catalytic residue eliminated MHF production in S. cerevisiae. Sequence analysis uncovered Cff1p homologs in many species, primarily bacterial and fungal, but also viral, archaeal, and higher eukaryotic. Cff1p homologs from organisms from all domains can complement a *cff1Δ* S. cerevisiae mutant and restore MHF production. In all cases tested, the identified catalytic residue is conserved and required for MHF to be produced. These findings increase the scope of possibilities for interdomain interactions via AI-2 and AI-2 mimics, highlighting the breadth of molecules and organisms that could participate in quorum sensing.

## Abstract (precis)

Quorum sensing is a cell-to-cell communication process that bacteria use to monitor local population density. Quorum sensing relies on extracellular signal molecules called autoinducers (AIs).

## INTRODUCTION

Bacteria use chemical communication to gauge local cell population density. This process, called quorum sensing, relies on the production, release, accumulation, and detection of extracellular signal molecules called autoinducers (AIs) (for recent reviews, see references 1–3). Quorum sensing enables bacteria to assess whether they are at a low or high cell density and, if the latter, engage in collective behaviors that, to be successful, require many cells acting in synchrony. For example, quorum sensing controls traits such as bioluminescence, biofilm formation, and virulence factor production.

The bioluminescent marine bacterium Vibrio harveyi is a model organism used to study quorum sensing. V. harveyi employs three AIs, namely, AI-1, CAI-1, and AI-2, that enable intraspecies, intragenera, and interspecies communication, respectively (4–7). Germane to this report is that AI-2 is bound by the receptor LuxP, and LuxP-AI-2 binding initiates a signal transduction cascade, the output of which is bioluminescence (5, 8, 9). AI-2 is produced and detected by diverse bacterial species (6, 10–12). Furthermore, human epithelial cells secrete an AI-2 mimic (here designated “mammalian AI-2 mimic”) of unknown structure that can be detected by LuxP, suggesting that the AI-2 signaling pathway could underpin interdomain communication (13).

**FIG 1:**
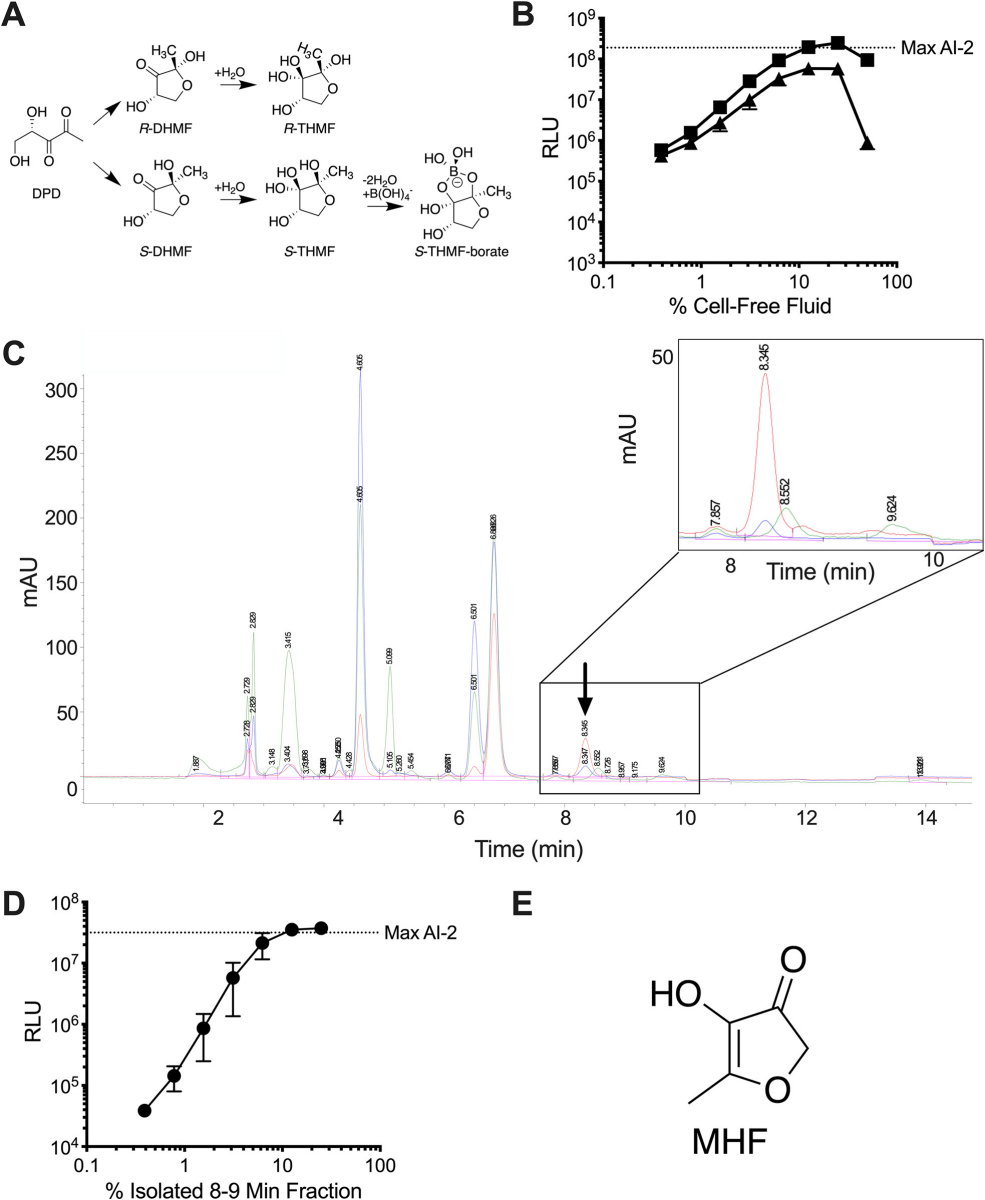
S. cerevisiae produces MHF, an AI-2 mimic. (A) Diagram showing the structure of DPD and relevant interconversions among molecules. (B) Light output by the V. harveyi TL-26 reporter strain in response to S. cerevisiae culture fluids containing yeast AI-2 mimic in PBS (squares) and in water (triangles). (C) Chromatogram depicting fractionation of yeast AI-2 mimic preparations. The area containing the active fraction is enlarged in the inset. The chromatograms show absorption at 214 (green), 254 (blue), and 280 (red) nm. The arrow depicts the peak containing the activity. mAU, milli-absorbance units. (D) Light output from the V. harveyi TL-26 reporter strain in response to a titration of the active 8- to 9-min fraction from C. (E) Structure of MHF. RLU denotes relative light units, which are bioluminescence/OD_600_ of the reporter strain, and the dotted line-labeled Max AI-2 refers to the activity from 125 nM DPD. In B and D, error bars represent standard deviations of biological replicates, *n* = 3.

Regarding AI-2 biosynthesis, the AI-2 precursor 4,5-dihydroxy-2,3-pentanedione (DPD) (Fig. 1A) is produced by the LuxS synthase from *S*-ribosylhomocysteine (SRH), a metabolic intermediate in *S*-adenosylmethionine (SAM)-dependent methylation pathways (6). DPD, the precursor to all AI-2 moieties, rapidly interconverts between different forms, and these rearranged structures can show preferences for binding to a particular bacterial quorum-sensing receptor. To activate the vibrio LuxP receptor, DPD must cyclize and coordinate borate to form the active AI-2 signaling moiety (2S,4S)-2-methyl-2,3,3,4-tetrahydroxytetrahydrofuran-borate (*S*-THMF-borate) (Fig. 1A) (8). The marine environment is borate rich, favoring formation of this final, borated signal molecule employed by V. harveyi and other vibrios. In boron-limited terrestrial environments, DPD rearranges to form (2R,4S)-2-methyl-2,3,3,4-tetrahydroxytetrahydrofuran (*R*-THMF), the active AI-2 moiety detected by enteric bacteria via a LuxP homolog called LsrB (Fig. 1A) (14). Another family of receptors, of which each contains a pCACHE domain, was recently discovered, expanding AI-2 detection mechanisms and the breadth of bacterial species that apparently respond to AI-2 (15).

Fungi also rely on quorum sensing to control behavior. In Saccharomyces cerevisiae, production of phenylethanol and tryptophol drives filamentous growth via activation of expression of *FLO11* encoding a glycoprotein required for flocculation and biofilm formation (16). Candida albicans uses two quorum-sensing molecules to regulate the transition from yeast to filamentous growth; tyrosol promotes the development of germ tubes required for hyphal growth, while farnesol inhibits this transition (17–20). Farnesol also mediates interdomain interactions by preventing toxin production by the bacterial pathogen Pseudomonas aeruginosa when in a mixed population with C. albicans (21, 22). Finally, farnesol is reported to modulate the human immune response in a C. albicans infection model (23–25). These findings hint at chemically mediated interdomain communication between fungi and other prokaryotic and eukaryotic organisms.

Here, we report a new interdomain quorum-sensing interaction. S. cerevisiae produces 4-hydroxy-5-methylfuran-3(2H)-one (MHF), a compound that mimics bacterial AI-2. Using V. harveyi as a reporter of AI-2 activity, we show that detection of and response to MHF require the LuxP receptor and signal transduction through the canonical V. harveyi quorum-sensing pathway. Screening of the S. cerevisiae deletion library revealed *CFF1* as a gene essential for MHF production. Cff1p, the protein encoded by the *CFF1* gene, is uncharacterized, but its crystal structure shows homology to sugar epimerases and isomerases (26). Mutation of a predicted catalytic residue, glutamic acid at position 44, eliminates MHF production by S. cerevisiae, suggesting that Cff1p may function as the MHF synthase. Many putative *CFF1* homologs exist in viral, archaeal, bacterial, fungal, and higher organismal genomes, and in the majority of cases we tested, the *CFF1* genes could complement a *cff1*Δ S. cerevisiae mutant and restore MHF production. Alignment of Cff1p homologs showed that the key glutamic acid residue is conserved, and in our test cases, it is required for activity. In summary, MHF has the ability to mimic AI-2, and MHF production may be prevalent in both prokaryotic and eukaryotic organisms. These findings highlight the expanding possibilities for interdomain signaling through AI-2 quorum-sensing pathways.

## RESULTS

### Purification and identification of MHF as an AI-2 mimic produced by S. cerevisiae.

We previously reported that human tissue culture cells of epithelial origin, when starved or subjected to tight junction disruption, produce a mimic of the bacterial quorum-sensing AI called AI-2 (13). These earlier findings inspired us to examine whether other eukaryotes produce AI-2 mimics. Here, we focused on the yeast S. cerevisiae for two reasons. First, evolutionarily, S. cerevisiae and humans diverged ∼1 billion years ago (27), possibly yielding insight into whether AI-2 mimic production does or does not occur widely across eukaryotes. Second, S. cerevisiae can be easily cultured, grows in virtually unlimited quantities, and survives in water, which are features predicted to accelerate purification and identification of interesting compounds (28). Relevant to this second point is that the identity of the mammalian AI-2 mimic remains unknown, primarily due to the inability to produce sufficient amounts for structural analyses. Moreover, the high salt conditions (phosphate-buffered saline [PBS]) required for mammalian AI-2 mimic production are incompatible with standard purification methods, such as high-performance liquid chromatography (HPLC).

We first tested whether S. cerevisiae makes a molecule that can mimic AI-2. S. cerevisiae MY8092 (hereafter called S. cerevisiae) was grown in synthetic defined (SD) medium with 2% glucose as the carbon source. Following 48 h of incubation, cell-free culture fluids were prepared and assessed for an activity capable of inducing light production in the V. harveyi AI-2 reporter strain called TL-26 (29). V. harveyi TL-26 produces maximum light in response to supplementation with 125 nM pure AI-2 (*S*-THMF-borate) (see Fig. S1A, dotted line, in the supplemental material). High-level activity was present in the S. cerevisiae cell-free culture fluids, suggesting that S. cerevisiae produces an AI-2 mimic (Fig. S1A).

10.1128/mBio.03303-20.1 FIG S1 S. cerevisiae isolates produce an AI-2 mimic. (A) Light output from the V. harveyi TL-26 reporter strain in response to cell-free fluids from S. cerevisiae grown for 48 h in synthetic defined medium with glucose as the carbon source. (B) V. harveyi TL-26 growth curves in the presence of 50% (green), 25% (red), 12.5% (blue), or 0% (black) AI-2 mimic. (C) As in B, with mimic samples supplemented with NaCl to a final concentration of 300 mM. (D) Light output from the V. harveyi TL-26 reporter strain in response to cell-free fluids from wild S. cerevisiae isolates grown to an OD_600_ of 25. The colors represent the S. cerevisiae isolates, as follows red, K1; orange, YJM308; yellow, YJM440; green, M32; cyan, YPS1000; blue, Y12; purple, Y2; and black, K11. (E) Light output from the V. harveyi TL-26 reporter strain in response to cell-free fluids from S. cerevisiae incubated at the designated OD_600_ values. (F) As in E, with the V. harveyi AI-1 reporter strain TL-25. RLU and Max AI-2 as in Fig. 1. Max AI-1 denotes activity from 500 nM AI-1. In all panels, error bars represent standard deviations of biological replicates, *n* = 3. Download FIG S1, TIF file, 1.8 MB. Copyright © 2021 Valastyan et al. 2021 Valastyan et al. https://creativecommons.org/licenses/by/4.0/ This content is distributed under the terms of the Creative Commons Attribution 4.0 International license.

Based on our finding that human epithelial cells produce the mammalian AI-2 mimic when starved in PBS, and again with the goal of facilitating purification, we next assessed AI-2 mimic production under starvation conditions. S. cerevisiae was grown to saturation in rich medium, washed twice, resuspended in either water or PBS, and incubated overnight at 30°C. AI-2 mimic activity was present in the collected fluids following resuspension of S. cerevisiae in both PBS (Fig. 1B, squares) and water (Fig. 1B, triangles). Addition of >25% (vol/vol) of the preparation made in water was toxic to V. harveyi TL-26 (Fig. S1B). Toxicity is due to V. harveyi sensitivity to low salt, as mimic fractions supplemented with NaCl were not toxic (Fig. S1C).

To discover whether AI-2 mimic production occurred broadly among wild yeasts or was restricted to laboratory S. cerevisiae, a panel of wild S. cerevisiae isolates obtained from different environments ranging from clinical settings to vineyards was tested for production of activity using the V. harveyi TL-26 reporter (Fig. S1D) (30, 31). All the production profiles mirrored that of laboratory S. cerevisiae, suggesting that the AI-2 mimic is broadly made by S. cerevisiae strains.

To garner sufficient yeast AI-2 mimic for structural analysis, we tested the limit to which we could concentrate the activity. At the final step of the above preparation procedure, the washed S. cerevisiae cells were resuspended in water at different cell densities, from an optical density at 600 nm (OD_600_) of 1 to 128. Following an overnight incubation, cell-free culture fluids were analyzed for activation of light production in V. harveyi TL-26. Yeast AI-2 mimic activity increased with increasing S. cerevisiae cell density (Fig. S1E). Moreover, the activity was specific to the AI-2 quorum-sensing pathway, as light production was not induced by the preparations when supplied to a V. harveyi reporter strain (TL-25) that is incapable of detecting AI-2 but, rather, responds exclusively to the V. harveyi quorum-sensing AI called 3-hydroxy-C4-homoserine lactone (AI-1) (29, 32) (Fig. S1F).

To identify the chemical structure of the yeast AI-2 mimic, washed S. cerevisiae cells were resuspended in water at an OD_600_ of 100. Following an overnight incubation, the cell-free fluids were collected and concentrated by lyophilization. Activity-guided HPLC fractionation on a Luna C_18_ reverse-phase column revealed one peak at 8.3 min that exhibited absorption at 254 nm (blue trace, Fig. 1C, arrow and inset) and 280 nm (red trace, Fig. 1C, arrow and inset). The material did not absorb significantly at 214 nm (green trace, Fig. 1C, arrow and inset). The peak contained high levels of yeast AI-2 mimic activity, as judged by the V. harveyi TL-26 reporter strain (Fig. 1D). We pooled this peak from multiple such column runs and prepared the sample for nuclear magnetic resonance (NMR) and mass spectral analyses as described in the Materials and Methods.

Identification of the bioactive molecule relied on a comparison of results from liquid chromatography-mass spectrometry (LC-MS), NMR, and gas chromatography (GC)-MS. LC-MS analysis showed that two components were present in the active fraction. From our initial HPLC fractionation, these components correspond to the peak with absorption at 254 nm and 280 nm (the active peak) and the peak with absorption at 214 nm (an inactive contaminant), which could not be completely separated for LC-MS. The bioactive component had an exact mass of 115.039 (M+H) and a putative molecular formula of C_5_H_6_O_3_. These data, combined with an analysis of key peaks in the ^13^C NMR spectra (signals at δ 194, 172, and 134 ppm), led us to consider structures A to D (see Fig. S2A in the supplemental material). Definitive evidence for structure A was obtained by GC-MS analysis, including matching of the fragmentation pattern of the active component against a database of known structures (Fig. S2B). The yeast AI-2 mimic structure A was identified as MHF (Fig. 1E, Fig. S2A). Indeed, a comparison of mass spectral, NMR, and HPLC analytical data confirmed that MHF purified from S. cerevisiae was identical to an authentic commercial sample of MHF (Fig. S2C and D).

10.1128/mBio.03303-20.2 FIG S2 Identification of the AI-2 mimic as MHF. (A) Compounds A to D proposed as candidate structures for the yeast AI-2 mimic. (B) GC-MS profile for the peak containing the active component from the yeast AI-2 mimic preparation. Inset, National Institute of Standards and Technology (NIST) database GC-MS spectrum for MHF. (C) ^1^H NMR (DMSO-*d_6_*) of commercial MHF. (D) ^13^C NMR (DMSO-*d_6_*) of commercial MHF. Download FIG S2, PDF file, 0.1 MB. Copyright © 2021 Valastyan et al. 2021 Valastyan et al. https://creativecommons.org/licenses/by/4.0/ This content is distributed under the terms of the Creative Commons Attribution 4.0 International license.

### The mammalian AI-2 mimic is not MHF.

With the MHF structure in hand, we could investigate whether the S. cerevisiae and the previously reported mammalian AI-2 mimic are identical or not. As noted earlier, the mammalian AI-2 mimic has not been identified, so we did not have purified compound (13). Rather, we made a preparation from Caco-2 cells containing high-level mammalian AI-2 mimic activity in PBS (13). To determine the elution pattern for MHF in such Caco-2 cell preparations, we spiked commercial MHF into the mammalian AI-2 mimic preparation prior to HPLC fractionation. MHF eluted at 14 min (see Fig. S3A, arrow, in the supplemental material) in the context of Caco-2 culture fluids. Samples from Caco-2 cells that had not been spiked did not have a peak at the expected elution time for MHF (Fig. S3B, arrow). To eliminate the possibility that MHF was present in the Caco-2 cell preparations but at a level below the UV detection limit on the HPLC instrument, we tested all of the fractions for activity in the V. harveyi TL-26 reporter assay. While the reporter assay showed that the mammalian AI-2 mimic was indeed present in the non-MHF-spiked Caco-2 preparations (Fig. S3C, black), there was no activity in the 12- to 14-min HPLC fraction (Fig. S3C, red). Collectively, these data demonstrate that the mammalian AI-2 mimic is not MHF. In future studies, we will focus on identification of the mammalian AI-2 mimic.

10.1128/mBio.03303-20.3 FIG S3 The mammalian AI-2 mimic is not MHF. (A) The mammalian AI-2 mimic preparation was fractionated by HPLC with a spike of 2 μg/ml of commercial MHF (arrow). (B) As in A but lacking the MHF spike. The arrow shows where MHF would elute if present. (C) Light output from the V. harveyi TL-26 reporter strain in response to the mammalian AI-2 mimic prior to HPLC fractionation (black) and activity from the 12- to 14-min fraction (red). In C, error bars represent standard deviations of biological replicates, *n* = 3. mAU = milli-absorbance units. RLU and Max AI-2 as in Fig. 1. Download FIG S3, TIF file, 1.2 MB. Copyright © 2021 Valastyan et al. 2021 Valastyan et al. https://creativecommons.org/licenses/by/4.0/ This content is distributed under the terms of the Creative Commons Attribution 4.0 International license.

### MHF agonizes LuxP with a Nanomolar EC_50_.

**FIG 2:**
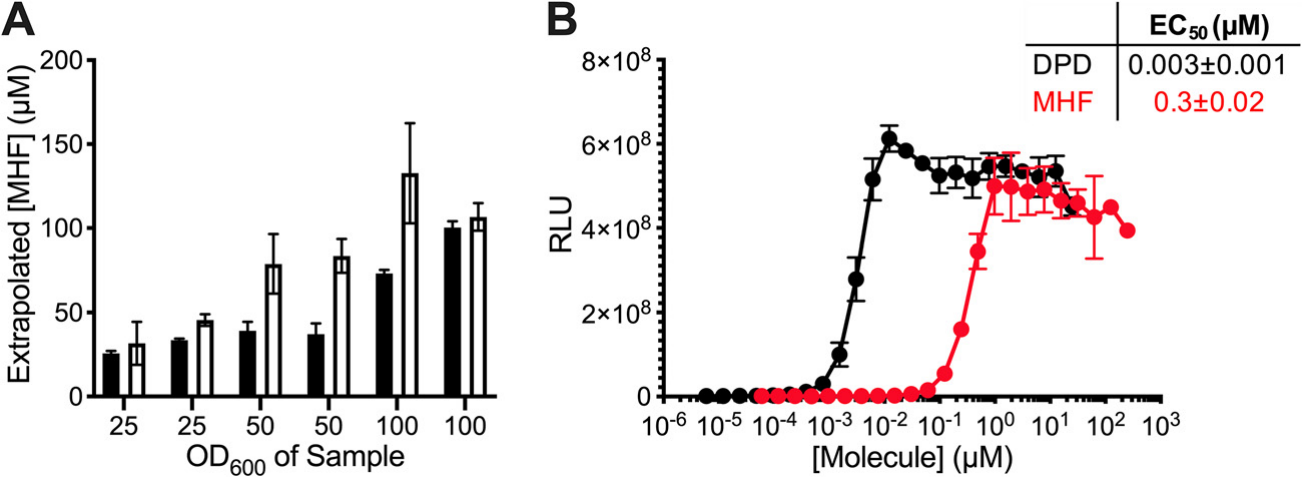
MHF agonizes the LuxP receptor with a nanomolar EC_50_. (A) Quantitation of MHF levels in yeast AI-2 mimic preparations from S. cerevisiae concentrated to OD_600_ of 25, 50, or 100 using integration under HPLC peaks (black) or activity from the V. harveyi TL-26 reporter strain (white). (B) Light output by the V. harveyi TL-26 reporter strain in response to DPD (black) or MHF (red). The table shows the EC_50_ values. RLU as in Fig. 1. In A, error bars represent standard deviations of technical replicates, *n* = 3. In B, error bars represent standard deviations of biological replicates, *n* = 3.

We next assessed the quantity of MHF present in S. cerevisiae cell-free fluids. To do this, we grew S. cerevisiae, pelleted and washed the cells, and then resuspended the cells at three different cell densities in water. Following overnight incubation, we removed the cells and compared the activities in each of the cell-free fluids to known quantities of the commercial MHF standard. We used two different MHF quantitation methods (Fig. 2A). First, different concentrations of commercial MHF were assessed by HPLC, and the areas under the MHF peaks were used to generate a standard curve. The S. cerevisiae preparations were likewise subjected to HPLC analysis, and the amount of MHF present in each sample was calculated by interpolating the area under the HPLC peak to that from the standard curve (Fig. 2A, black bars). Second, commercial MHF was assessed in the V. harveyi TL-26 reporter assay at different concentrations to generate an activity-based standard curve. The S. cerevisiae cell-free fluids were identically assayed, and the MHF concentration in each preparation was estimated from the activity standard curve (Fig. 2A, white bars). The concentrations of MHF in the preparations calculated by the two methods were in close agreement. Assuming MHF production has a linear relationship with OD_600_ values, we can use our data to estimate that S. cerevisiae produced 1.2 ± 0.4 μM MHF per OD_600_ of cells. In the context of detection by the V. harveyi quorum-sensing apparatus, the 50% effective concentration (EC_50_) for AI-2 is 3 nM and that for MHF is 300 nM (Fig. 2B). Thus, while both compounds exert activity in this system within the concentration range reported for bacterial AIs (33–36), the LuxP receptor prefers AI-2 over MHF.

### Identification of *CFF1* as an S. cerevisiae gene essential for MHF production.

**FIG 3:**
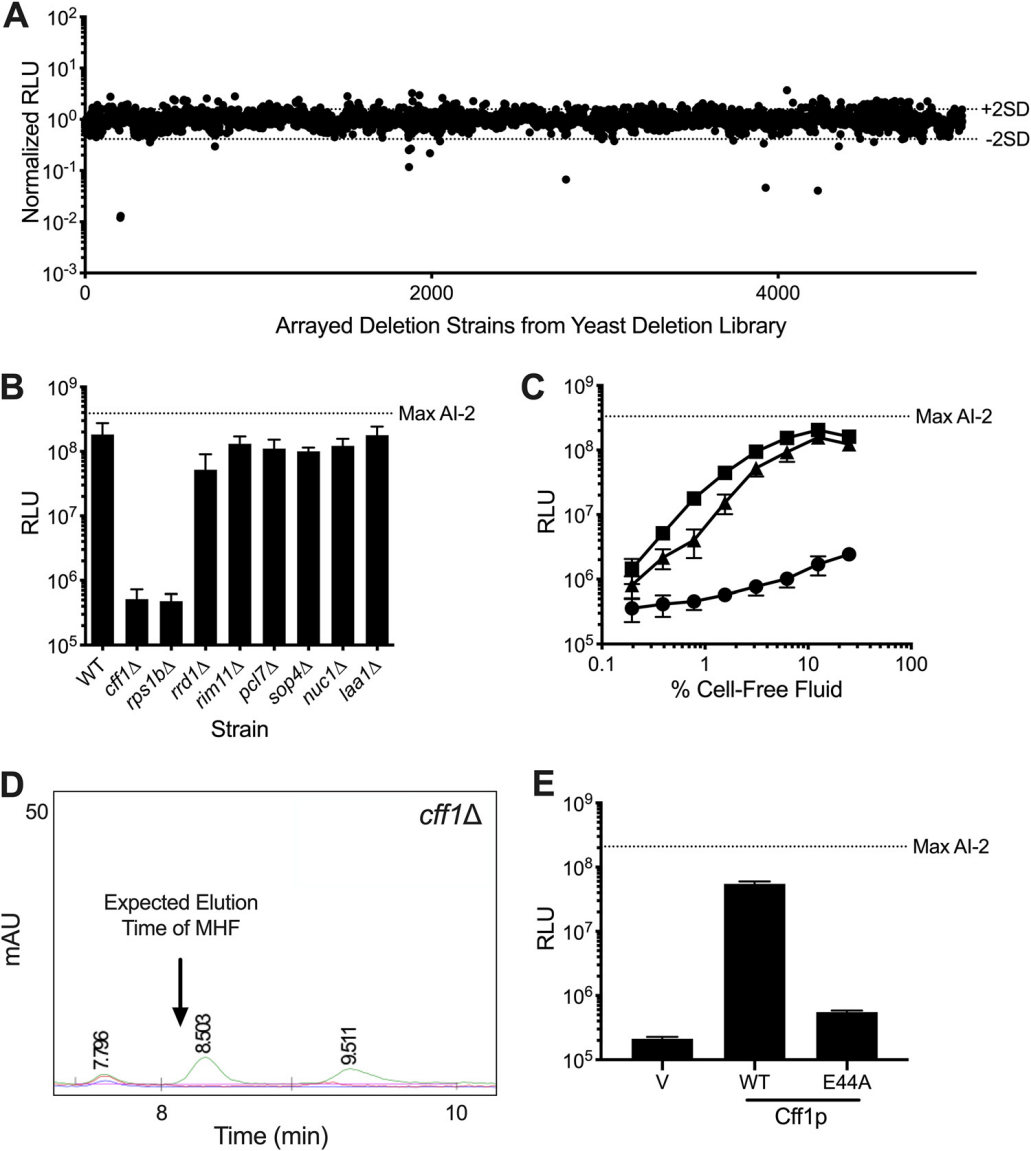
Cff1p is required for S. cerevisiae to produce MHF. (A) Normalized light output from the V. harveyi TL-26 reporter strain in response to culture fluids from the mutants in the S. cerevisiae deletion library. Each point represents the reporter response to a fluid made from a unique yeast mutant. Dotted lines labeled +2SD and −2SD show two standard deviations above and below the mean, respectively. (B) Light output from the V. harveyi TL-26 reporter strain in response to culture fluids from the putative hit S. cerevisiae mutants from A. (C) Light output from the V. harveyi TL-26 reporter strain in response to culture fluids from WT S. cerevisiae (squares) and the *cff1Δ* (circles) and *rps1bΔ* (triangles) mutants. (D) Portion of an HPLC trace from fractionation of yeast AI-2 mimic preparation made from *cff1Δ* S. cerevisiae. The chromatograms show absorption at 214 (green), 254 (blue), and 280 (red) nm. The arrow shows the expected elution time for MHF based on WT S. cerevisiae results (see Fig. 1C). (E) Light output from the V. harveyi TL-26 reporter strain in response to cell-free fluids made from *cff1Δ* S. cerevisiae that produced either a HALO control (designated “V”), Cff1p-HALO (designated WT), or Cff1p-E44A-HALO (designated E44A). Normalized RLU in A are RLU of the given sample divided by the average RLU from all plates assayed on a single day. RLU and Max AI-2 as in Fig. 1. In B, C, and E, error bars represent standard deviations of biological replicates, *n* = 3. In B and E, 10% (vol/vol) of cell-free fluid was added in each case.

To identify the component(s) responsible for MHF production in S. cerevisiae, we screened the yeast deletion library for an S. cerevisiae mutant that was defective in MHF production (37–39). As described in the Materials and Methods, cell-free fluid preparations were made from >5,000 S. cerevisiae mutants and incubated with the V. harveyi TL-26 reporter strain. Bioluminescence was measured to assess the ability of each S. cerevisiae mutant to make MHF (Fig. 3A). Mutants were identified that elicited at least two standard deviations less light from the reporter strain than the mean amount of light production elicited from all strains (Fig. 3A). Eight putative mutants were retested for the ability to make activity (Fig. 3B). Two mutants, namely, *cff1Δ* and *rps1bΔ*, failed to activate the reporter strain. Cff1p (systematic name, YML079wp) is a cupin superfamily protein (26). Rps1bp (systematic name, YML063wp) is a component of the 40S ribosomal subunit (40). The six other potential mutants proved to have been false positives upon reassessment (Fig. 3B).

To verify the phenotypes of the mutants, clean deletions of *CFF1* and *RPS1B* were constructed in S. cerevisiae. The *cff1*Δ mutant displayed no defect in growth rate (see Fig. S4A, circles, in the supplemental material; compare to wild-type [WT] growth shown by the squares). As previously reported (41), the *rps1bΔ* mutant had a growth defect (Fig. S4A, triangles). Neither mutant exhibited sensitivity to overnight incubation in water (Fig. S4B). Importantly, culture fluids prepared from the clean *rps1bΔ* mutant produced nearly the wild-type level of AI-2 mimic activity, as determined by the ability to induce light production in the V. harveyi TL-26 reporter (Fig. 3C, triangles). In contrast, preparations made from the *cff1Δ* mutant had no activity (Fig. 3C, circles). PCR analysis revealed that the mutant annotated as *rps1bΔ* in the yeast deletion library, in fact, possesses a deletion in *CFF1*, explaining its inability to stimulate the reporter strain as well as the ability of our newly constructed *rps1bΔ* mutant to produce activity. Thus, *CFF1* is the only gene revealed by our screen to be required for production of the activity we are monitoring.

10.1128/mBio.03303-20.4 FIG S4 Growth and survival of S. cerevisiae mutants, their MHF production profiles, and Cff1p abundance. (A) Growth curves of WT S. cerevisiae (squares), *cff1Δ* S. cerevisiae (circles), and *rps1bΔ* S. cerevisiae (triangles). OD_600_ was monitored every 20 min. (B) Survival of the strains in A following overnight incubation in water at 30°C. Change in CFU is calculated as CFU after incubation divided by CFU before incubation. (C) Light output by the V. harveyi TL-26 reporter strain in response to activity present in the 8- to 9-min HPLC fraction from an AI-2 mimic preparation made from *cff1Δ* S. cerevisiae. (D) Levels of the designated Cff1p-HALO proteins visualized by staining with HaloTag TMR ligand. The loading control shows a prominent band from Coomassie staining of the gel. RLU and Max AI-2 as in Fig. 1. In A, B, and C, error bars represent standard deviations of biological replicates, *n* = 3. Download FIG S4, TIF file, 1.0 MB. Copyright © 2021 Valastyan et al. 2021 Valastyan et al. https://creativecommons.org/licenses/by/4.0/ This content is distributed under the terms of the Creative Commons Attribution 4.0 International license.

To confirm that the yeast AI-2 mimic activity produced by the protein encoded by *CFF1* is MHF, we prepared and fractionated cell-free culture fluids from the *cff1Δ* strain using the identical procedure we used for isolation of MHF from wild-type S. cerevisiae. No MHF peak could be detected in the *cff1Δ* mutant preparation (Fig. 3D; compare to Fig. 1C, inset). Consistent with this finding, the relevant HPLC column fraction had no activity in the V. harveyi TL-26 reporter assay (Fig. S4C). These data suggest that Cff1p has a required role in MHF biosynthesis in yeast.

Our finding that Cff1p is required for MHF production is surprising. The presence of MHF in fermented food products made from S. cerevisiae has been reported; however, the suggested route to MHF is either spontaneous starting from d-ribulose-5-phosphate (42–45) or under extreme conditions, via the Maillard reaction (46, 47). Quite to the contrary, our data suggest that MHF production in S. cerevisiae is enzyme catalyzed and under physiological conditions. Cff1p has not been characterized. However, there does exist a crystal structure (26). It shows a putative ligand binding pocket containing amino acid residues identical to those required for catalysis by epimerases and isomerases that share the cupin fold (48). Specifically, the conserved E44 residue is proposed to have a catalytic role. We made an E44A substitution in Cff1p and assayed the mutant protein for MHF production. The substitution did not alter Cff1p stability as judged by visualization of a fused HALO tag (Fig. S4D); however, culture fluids prepared from the S. cerevisiae Cff1p-E44A mutant elicited 100-fold less light from the V. harveyi TL-26 reporter strain than preparations from WT S. cerevisiae (i.e., less than 1% activity remained) (Fig. 3E), showing that the glutamate residue at position 44 is key for the presumptive enzymatic activity that generates MHF.

### *CFF1* homologs exist in organisms from all domains.

**FIG 4:**
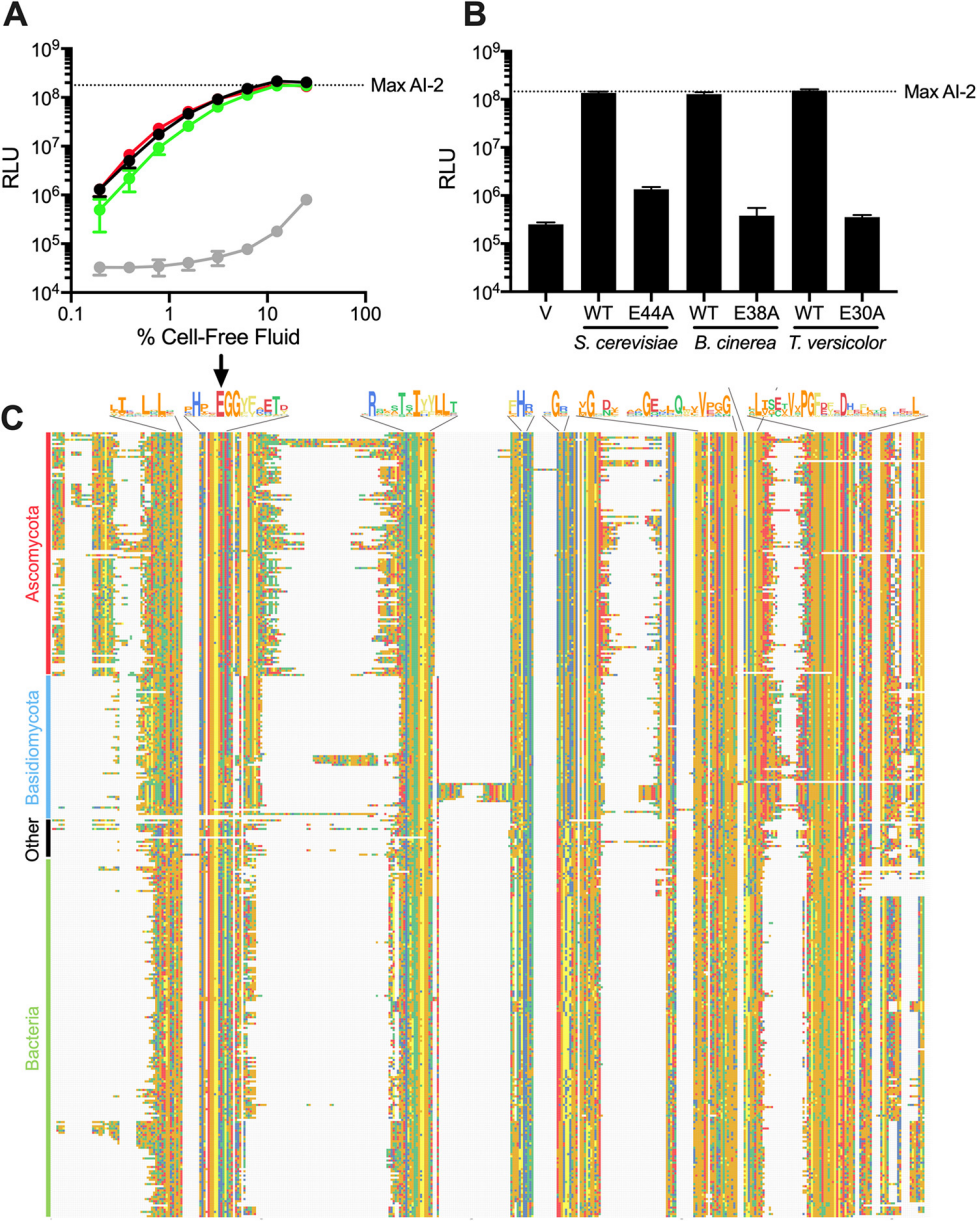
Cff1p homologs restore MHF production to *cff1Δ* S. cerevisiae. (A) Light output from the V. harveyi TL-26 reporter strain in response to cell-free fluids from *cff1Δ* S. cerevisiae expressing *CFF1* homologs from *B. cinerea* (green), *T. versicolor* (red), S. cerevisiae (black), and a vector control (gray). (B) Light output from the V. harveyi TL-26 reporter strain in response to cell-free fluids prepared from *cff1Δ* S. cerevisiae carrying the designated *CFF1* homologs and alleles. The vector control is designated V. Cell-free fluids were added at 10% (vol/vol). (C) Alignment of putative Cff1p homologs, trimmed to the first and final amino acids of S. cerevisiae Cff1p. Only one species per genus is shown. Alignment was performed using Clustal Omega (79). Negatively charged residues are shown in red, small hydrophobic residues in orange, aromatic hydrophobic residues in yellow, polar uncharged residues in green, and positively charged residues in blue. Shown above the alignment are consensus sequences for regions containing amino acids that are conserved in >75% of the aligned proteins. Arrow designates location of conserved glutamate residue. RLU and Max AI-2 as in Fig. 1. In A and B, error bars represent standard deviations of biological replicates, *n* = 3.

Cff1p has structural similarity to sugar isomerases and epimerases, and the Cff1p amino acid sequence is similar to proteins of unknown function (26). Recently, Tourneroche et al. reported 12 wild fungal species that exist as endomicrobiota of kelp and that possess AI-2 activity, as judged by a V. harveyi reporter system analogous to the one we use here (49). The molecule(s) responsible for the AI-2 activity have not been identified. We wondered whether these fungal species might make MHF. Examination of their proteomes revealed Cff1p homologs in 2 of the 12 species, namely, Trametes versicolor and Botrytis cinerea, with approximately 50% similarity and 35% identity, respectively, to S. cerevisiae Cff1p at the amino acid sequence level (see Fig. S5A in the supplemental material). Both of these species’ Cff1p homologs have high conservation in the putative ligand binding domain, and they each possess a residue equivalent to E44 in S. cerevisiae Cff1p (Fig. S5A, arrow). To test for function, we cloned these two genes and introduced them into our *cff1Δ* S. cerevisiae mutant under the control of the native S. cerevisiae *CFF1* promoter. Unlike the *cff1Δ* S. cerevisiae mutant that produced no MHF, the mutant carrying each homolog produced activity sufficient to induce maximal light production in the V. harveyi TL-26 reporter strain (Fig. 4A). The E38A and E30A substitutions in the *B. cinerea* and *T. versicolor* Cff1p proteins (equivalent to E44A in S. cerevisiae Cff1p), respectively, eliminated production of the activity (Fig. 4B). In both cases, the WT and mutant proteins were made at approximately the same levels and were equally stable (Fig. S5B). HPLC fractionation confirmed that MHF was indeed produced in *cff1Δ* S. cerevisiae carrying the WT *CFF1* homologs, and no MHF could be detected in the cases in which mutant *CFF1* alleles were present (see Fig. S6 in the supplemental material). Thus, both the *T. versicolor* and *B. cinerea CFF1* genes can complement the *cff1Δ* S. cerevisiae defect and restore MHF production, and a glutamate at a position equivalent to 44 in S. cerevisiae Cff1p is required. In the cases of the other 10 fungi that Tourneroche et al. reported to possess AI-2 activity (49), we do not know whether they make a different active molecule or, alternatively, if they possess Cff1p proteins that are unrecognizable through the database search we performed.

10.1128/mBio.03303-20.5 FIG S5 Comparison of Cff1p homologs and their abundances in *cff1Δ* S. cerevisiae. (A) Amino acid sequence alignment of Cff1p homologs from S. cerevisiae, *B. cinerea*, and *T. versicolor*. Black and gray boxes designate amino acid identity and similarity, respectively. The residues equivalent to E44 in S. cerevisiae are shown by the arrow. (B) Protein gel showing levels of WT and mutant Cff1p-HALO from S. cerevisiae, *B. cinerea*, and *T. versicolor* produced by *cff1Δ* S. cerevisiae carrying the corresponding genes. Cff1p-HALO and HALO bands are labeled. Additional bands, likely protein degradation products, are present. The loading control is a prominent band from Coomassie staining of the gel. Download FIG S5, TIF file, 2.1 MB. Copyright © 2021 Valastyan et al. 2021 Valastyan et al. https://creativecommons.org/licenses/by/4.0/ This content is distributed under the terms of the Creative Commons Attribution 4.0 International license.

10.1128/mBio.03303-20.6 FIG S6 Homologs of Cff1p can complement the loss of MHF production in *cff1Δ* S. cerevisiae. (A) Chromatogram depicting HPLC fractionation of yeast AI-2 mimic prepared from *cff1Δ* S. cerevisiae carrying an empty vector (denoted V) (inset) or supplemented with 125 ng exogenous MHF. The chromatograms show absorption at 214 (red), 254 (blue), and 280 (green) nm. The boxed region shows that MHF absorbs at 254 (blue) and 280 (green) nm and elutes at 6.448 min. This region should be compared with that in the inset showing that this peak is absent in the same sample that did not receive exogenous MHF. (B) Segments of chromatograms showing the same regions boxed in A for fractionations of yeast AI-2 mimic prepared from *cff1Δ* S. cerevisiae carrying WT and the designated mutant *CFF1* alleles from S. cerevisiae, *B. cinerea*, and *T. versicolor*. The arrows in the top row of boxes denote the MHF peaks in the samples prepared from S. cerevisiae carrying each WT *CFF1* gene. The bottom row of boxes show that those peaks are absent when S. cerevisiae carries the mutant genes. In all panels, absorption at 214 nm is shown in red, 254 nm is shown in blue, and at 280 nm is shown in green. Note that the color scheme differs from other figures with HPLC traces. mAU, milli-absorbance units. Download FIG S6, TIF file, 1.4 MB. Copyright © 2021 Valastyan et al. 2021 Valastyan et al. https://creativecommons.org/licenses/by/4.0/ This content is distributed under the terms of the Creative Commons Attribution 4.0 International license.

A search of the nonredundant protein sequence database (50) for proteins with homology to S. cerevisiae, *T. versicolor*, and *B. cinerea* Cff1p uncovered three additional *Saccharomyces* species possessing proteins with an average identity of 90% to Cff1p. More broadly, we identified 410 non-*Saccharomyces* fungal species possessing proteins harboring 26% to 71% identity to Cff1p. Included were members of the Ascomycota phylum, such as Aspergillus fumigatus and Neurospora crassa, and the Basidiomycota phyla, such as Cryptococcus neoformans. We also identified >350 prokaryotes possessing putative proteins with 25% to 45% identity to Cff1p. These species exist in 11 phyla and include multiple pseudomonads, staphylococci, and bacilli. Our search also uncovered 25 other organisms, spanning all domains, with potential Cff1p homologs. The majority of these organisms exist in the marine environment, for example, the acorn worm Saccoglossus kowalevskii, the green alga Ostreococcus lucimarinus, and the archaeon Methanohalophilus halophilus (Fig. 4C; see Fig. S7 in the supplemental material). At present, we do not know whether this representation indicates a dominant biological function in the marine niche or if it stems from biased sampling. Notably, E44 is conserved in 97% of these potential homologs (Fig. 4C, arrow).

10.1128/mBio.03303-20.7 FIG S7 Organisms from all domains contain Cff1p homologs—expanded analysis. Phylogenetic tree showing all genera with Cff1p homologs uncovered by BLASTp analysis (50). Red, fungal species belonging to the Ascomycota phylum; blue, fungal species belonging to the Basidiomycota phylum; green, bacterial species; black, all other species. The frequencies of the two highest level nodes, following 500 bootstrap replications, are shown. Download FIG S7, TIF file, 1.4 MB. Copyright © 2021 Valastyan et al. 2021 Valastyan et al. https://creativecommons.org/licenses/by/4.0/ This content is distributed under the terms of the Creative Commons Attribution 4.0 International license.

**FIG 5:**
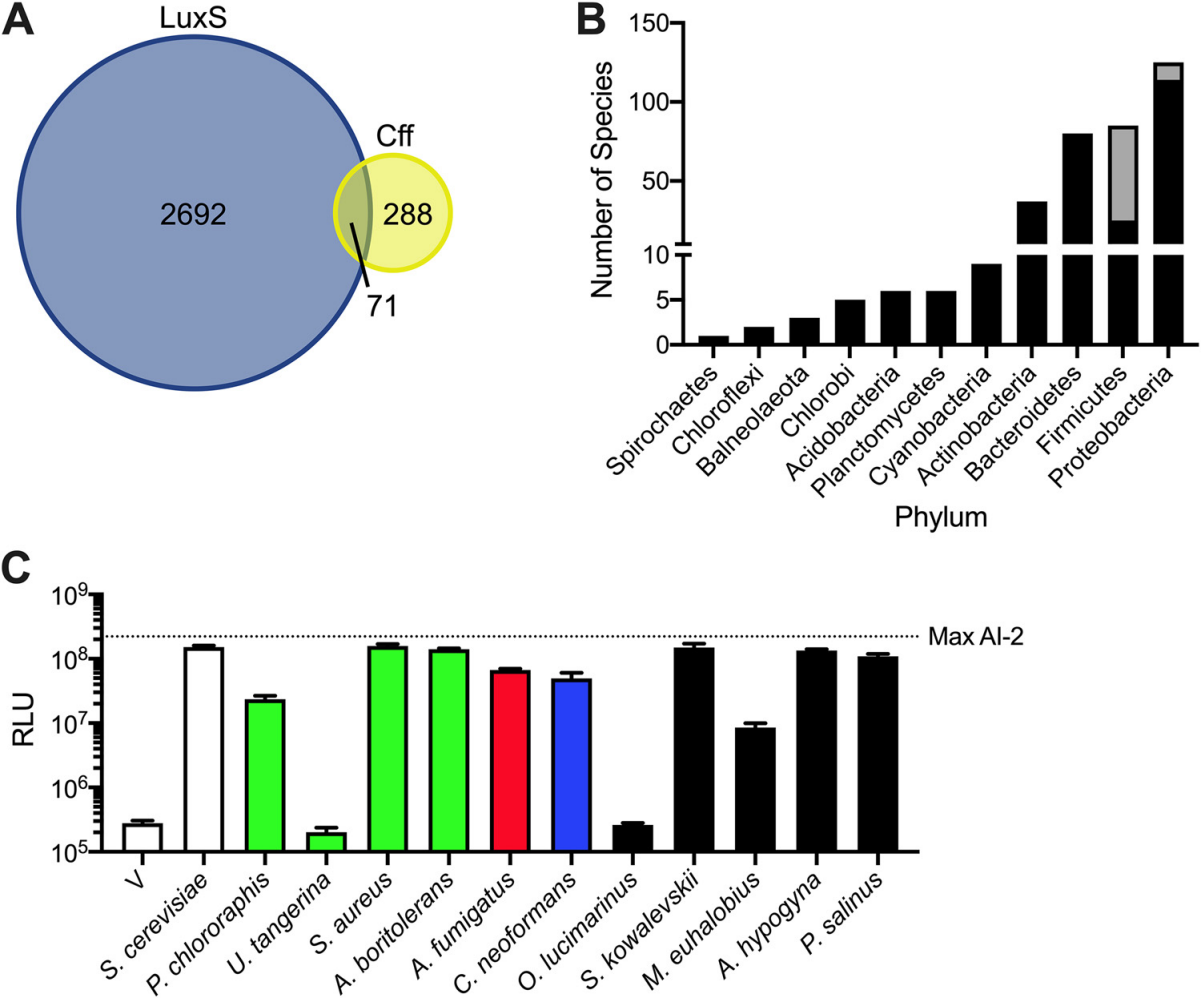
Organisms from all domains contain Cff1p homologs. (A) Venn diagram displaying the numbers of bacterial species containing LuxS and/or Cff homologs. (B) Bacterial phylogeny distribution showing species containing potential Cff homologs. Black bars represent species possessing only Cff. Gray bars represent species possessing both LuxS and Cff. (C) Light output from the V. harveyi TL-26 reporter strain in response to cell-free fluids prepared from S. cerevisiae expressing *CFF1* and *cff* homologs. Bars are colored according to the groups in Fig. 4C and Fig. S7, as follows: green, *Bacteria*; red, Ascomycota; blue, Basidiomycota; black, other. The vector control is designated V. Cell-free fluids were added at 10% (vol/vol). RLU and Max AI-2 are as in Fig. 1. In C, error bars represent standard deviations of biological replicates, *n* = 3.

It is intriguing that bacterial species may possess *cff* genes (note that we designate the bacterial genes and proteins as *cff* and Cff, respectively, and the fungal genes and proteins *CFF1* and Cff1p, respectively). Thousands of bacterial species are known to synthesize the interspecies quorum-sensing AI AI-2 via possession of *luxS* encoding the AI-2 synthase (10). To investigate whether bacteria could potentially make both MHF and AI-2, we performed database analyses. According to the nonredundant protein sequences database (50), only ∼20% of bacterial species possessing a *cff* homolog also harbor *luxS* (Fig. 5A). The majority of bacteria that possess both *luxS* and *cff* genes belong to the *Firmicutes* phylum (Fig. 5B), a phylum considered ancestral to other bacterial phyla. This pattern suggests that a shared ancestor possessed both genes and that, generally, the species maintained only one of the two genes as they diverged.

To test whether additional Cff and Cff1p homologs are functional, we selected 10 additional organisms, namely, four prokaryotes (Pseudomonas chlororaphis, Umezawaea tangerina, Staphylococcus aureus, and Algoriphagus boritolerans), two fungi (A. fumigatus and C. neoformans), and four organisms from other kingdoms (O. lucimarinus, S. kowalevskii, M. euhalobius, and Achlya hypogyna) that possess putative Cff or Cff1p proteins (Table 1). Our choices included clinically relevant organisms (i.e., S. aureus and C. neoformans) and marine organisms (i.e., *O. lucimarinus* and *S. kowalevskii*) that may coexist with bacteria that use AI-2-LuxP-mediated quorum sensing. Additionally, we tested proteins from organisms representing unique phyla and proteins with various levels of amino acid identity relative to S. cerevisiae Cff1p (Table 1). To assay activity, we expressed the candidate genes in our *cff1Δ* S. cerevisiae strain under the control of the endogenous S. cerevisiae *CFF1* promoter. All of the homologs except those from *U. tangerina* and *O. lucimarinus* complemented the loss of Cff1p in S. cerevisiae, driving sufficient MHF production to induce light production in the V. harveyi TL-26 reporter strain (Fig. 5C). This result suggests that these, and likely many other, organisms harbor the potential to synthesize MHF.

**TABLE 1:** Cff1p homology in select species

Organism by group	Species	% Coverage	% Identity	Complements *cff1Δ* S. cerevisiae?
Bacteria				
*Proteobacteria*	Pseudomonas chlororaphis	76	43	Yes
*Actinobacteria*	Umezawaea tangerina	77	37	No
*Firmicutes*	Staphylococcus aureus	73	32	Yes
*Bacteroides*	Algoriphagus boritolerans	77	28	Yes
Fungi				
Ascomycota	*Botrytis cinerea*	89	44	Yes
Basidiomycota	*Trametes versicolor*	76	42	Yes
Ascomycota	Aspergillus fumigatus	93	40	Yes
Basidiomycota	Cryptococcus neoformans	87	35	Yes
Other				
Algae	*Ostreococcus lucimarinus*	68	41	No
Animal	*Saccoglossus kowalevskii*	79	38	Yes
Archaea	*Methanohalophilus euhalobius*	76	32	Yes
Protista	*Achlya hypogyna*	69	31	Yes
Virus	*Pandoravirus salinus*	69	24	Yes

Compared to BLASTp, Interpro (51) provides a dramatically larger set of proteins under the class “uncharacterized protein, YML079W-like” (i.e., the S. cerevisiae systematic name for Cff1p). This group contains proteins from >3,000 different organisms, including >2,400 species of bacteria. Notably, 95% of these putative Cff homologs have the conserved glutamate at the position corresponding to 44 in the S. cerevisiae Cff1p. Thus, the Interpro data suggest that, potentially, the number of bacterial species containing *cff* is on the same scale as those possessing *luxS*, as Interpro lists ∼2,500 different species (primarily bacterial) that contain *luxS*. In this data set, ∼15% of the bacterial species contain both *luxS* and *cff*.

We were especially intrigued that the Interpro database search revealed a virus with a potential Cff homolog, Pandoravirus salinus. Using the above strategy, we tested the functionality of the *P. salinus* Cff homolog and found that, indeed, the viral *cff* gene complements the loss of *CFF1* in S. cerevisiae (Fig. 5C). This finding provides initial validation for the output of the larger Interpro data set and presages the existence of many additional functional Cff1p homologs. Moreover, this final result, coupled with the other findings here, shows that MHF production could occur across the archaeal, bacterial, and eukaryotic domains, as well as among viruses.

## DISCUSSION

Quorum sensing is the process by which bacteria monitor their local cell population density and determine when it is appropriate to engage in collective behaviors (1–3). Bacteria often employ multiple AIs, encoding distinct information about species relatedness, which presumably enables them to take a census of “self” and “other.” The AI-2-LuxP quorum-sensing pathway is proposed to be used for the latter, to monitor the total cell density of the vicinal community. Previously, we showed that mammalian cells can make a mimic of AI-2 that activates quorum sensing via LuxP, providing a founding example of interdomain quorum-sensing-mediated communication (13). Here, we show that S. cerevisiae, another eukaryotic organism, makes MHF, which can induce quorum-sensing behavior through the canonical AI-2 pathway. Our preliminary studies suggest that MHF production may be widespread across domains. Presumably, the ability of MHF to substitute for AI-2 enables bacteria that possess LuxP to detect the presence of other bacterial cells, higher organisms, and possibly viruses in the environment.

The mammalian AI-2 mimic is distinct from MHF, suggesting that multiple compounds might be exploited for AI-2-like interactions between eukaryotes and bacteria. Plants, insects, and fungi have previously been shown to produce MHF (42–44, 52–54), and based on the findings presented here, it is possible that MHF could be widely used for cross-domain interactions between bacteria and fungi. While future studies are necessary to understand the ecological significance of MHF in such presumptive interdomain interactions and, more specifically, what, if any, traits are controlled by MHF in organisms that produce MHF, it is already known that cross-communication between eukaryotes and prokaryotes can shape each participant’s biology. For example, quorum-sensing pathways drive behavior across domains in both mutualistic and parasitic relationships. Examples include communication between the gut and the microbiome (55), competition between C. albicans and P. aeruginosa during infection of the lung (56), and the ability of Legionella pneumophila to alter eukaryotic host cell migration (57). Beyond bacteria and eukaryotes, recent work demonstrates that quorum-sensing-mediated cross-communication occurs between bacteria and phages (58–60). Specifically, an AI called 3,5-dimethylpyrazin-2-ol (DPO) that is produced by many species of bacteria is detected by a phage that uses the information encoded in DPO to determine whether to enter the lytic or the lysogenic state (59). Our present work provides evidence for a virus with the potential to make MHF. Presumably, interdomain chemical interactions could enable sharing of and cheating on public goods, could enhance symbiosis or predation, or could allow particular organisms to coestablish niches in otherwise inhospitable environments. Notably, bacteria that synthesize AI-2 generally use it to regulate their own quorum-sensing-controlled behaviors (61). We do not yet know if S. cerevisiae and other eukaryotes use MHF as a quorum-sensing signal. Our intention now is to perform transcriptome sequencing (RNA-seq) on *cff1Δ* S. cerevisiae in the presence and absence of exogenously supplied MHF to discover the endogenous response.

MHF is a volatile compound that is used as a flavorant. Food scientists have previously shown that low levels of MHF exist in fermented foods, such as soy sauce and malt (62–64). As alluded to above, MHF is hypothesized to form spontaneously from pentose sugars that undergo the Maillard reaction (i.e., during cooking) or as a by-product (in fungi). Notably, MHF is also a breakdown product of DPD; however, S. cerevisiae lacks LuxS, so DPD is an unlikely source of MHF in fungi (44, 65). In the fungus Zygosaccharomyces rouxii, it is proposed that, following production of ribulose-5-phosphate by the pentose phosphate pathway, MHF forms spontaneously as a breakdown product (42). Here, we show that Cff1p, which is presumably an enzyme, is required for MHF production in S. cerevisiae. Z. rouxii contains a *CFF1* homolog, which suggested to us that the pathway we discovered here could be relevant in this organism. Indeed, cell-free culture fluids from WT Z. rouxii possess activity capable of inducing light production in V. harveyi TL-26, while reporter fluids prepared from a Z. rouxii *cff1Δ* strain are devoid of that activity (see Fig. S8 in the supplemental material).

10.1128/mBio.03303-20.8 FIG S8 Z. rouxii produce an AI-2 mimic and production requires *CFF1*. Light output from the V. harveyi TL-26 reporter strain in response to cell-free fluids from Z. rouxii (squares) and a Z. rouxii *cff1*Δ mutant (circles). RLU and Max AI-2 as in Fig. 1. Error bars represent standard deviations of biological replicates, *n* = 3. Download FIG S8, TIF file, 0.3 MB. Copyright © 2021 Valastyan et al. 2021 Valastyan et al. https://creativecommons.org/licenses/by/4.0/ This content is distributed under the terms of the Creative Commons Attribution 4.0 International license.

S. cerevisiae *CFF1* is a gene of unknown function. The crystal structure of S. cerevisiae Cff1p has been solved and shows similarity to sugar epimerases and isomerases (26). The Cff1p protein, like other cupins, contains a jelly-roll fold similar to those in germin- and auxin-binding proteins. In Cff1p, the regions adjacent to the cupin motif are distinct from previously studied members of this protein superfamily. Cff1p has notable similarity to the Salmonella enterica serovar Typhimurium protein RmlC, which catalyzes the conversion of dTDP-6-deoxy-d-xylo-4-hexulose to dTDP-6-deoxy-l-lyxo-hexulose (66). The crystal structure of RmlC bound to a substrate analog has been compared with that of S. cerevisiae Cff1p and shows that Cff1p possesses a binding pocket that could accommodate both the nucleotide with which Cff1p was cocrystallized and a sugar moiety. However, no sugar was present in the Cff1p crystal. The authors hypothesized that the pocket may bind a sugar nucleotide. Accordingly, the jelly-roll fold motif is shared with enzymes, such as phosphoglucose isomerase and dTDP-4-keto-6-deoxy-d-hexulose-3,5-epimerase (26). Given this relatedness and the fact that DPD, the nonborated precursor to AI-2, is a sugar, we suspect that Cff1p could be the synthase for MHF and that MHF is made from a sugar substrate.

Our study suggests that the scope of organisms that can participate in quorum sensing through AI-2-type pathways continues to increase, hinting that AI-2 quorum sensing mediates interspecies bacterial communication and interdomain communication between bacteria and eukaryotes and possibly viruses. Bacteria can distinguish among closely related quorum-sensing AIs, and they are capable of decoding and integrating the information contained in blends of AIs to drive appropriate behaviors based on the cell density and the species identities of neighboring bacteria. Cooccurrences of organisms from different domains have important ecological and medical implications (67–70). We highlight a few examples involving S. cerevisiae, the main focus of the present work; the presence of S. cerevisiae improves the ability of Pseudomonas putida to grow in glucose-containing medium (71), enhances the growth of lactic acid bacteria in nitrogen-rich environments (72), and stimulates the growth of multiple Acinetobacter species through the secretion of ethanol (73). Our future work will focus on how domain-spanning quorum-sensing cross-communication influences the behavior of the various participants and affects global community structures and their functioning.

## MATERIALS AND METHODS

### Strains, plasmids, and media.

Strains and plasmids used in this work are provided in Table S1 and S2, respectively, in the supplemental material. *YML079W* (*CFF1*) and *YML063W* (*RPS1B*) were deleted from S. cerevisiae using the standard kanMX insertion technique (38, 74). Geneticin (G418 sulfate) (Thermo Fisher Scientific) was added at 200 μg/ml for KanMX selection. S. cerevisiae was grown in yeast extract-peptone-dextrose (YPD) medium, unless specified otherwise (Thermo Fisher Scientific). V. harveyi was grown in Luria-Marine (LM) medium, and AI-2 reporter assays were performed in autoinducer bioassay (AB) medium (29). To generate pRS416-*CFF1*, *CFF1* and ∼150 bp upstream and downstream were cloned into the XhoI and XbaI (New England BioLabs) sites of pRS416 using standard cloning procedures. We tested two constructs, one containing the *CFF1* gene and 500 bp of upstream and downstream DNA and one containing the *CFF1* gene with upstream and downstream regions of ∼150 bp and encompassing only intergenic sequences between *CFF1* and its neighboring genes. Both constructs drove production of the same amount of MHF, suggesting that all of the necessary promoter and terminator elements for *CFF1* are in the intergenic regions. Therefore, we used the construct containing *CFF1* and only the intergenic regions for the work reported here. DNA encoding the *HALO* sequence was fused to that encoding the 3′ terminus of *CFF1* using Gibson assembly (New England BioLabs) (75). DNA encoding *CFF1* homologs was codon optimized and synthesized (Integrated DNA Technologies) before being inserted between the upstream and downstream regions of S. cerevisiae *CFF1*, cloned into pRS416 (76), and HALO tagged using the strategy outlined above or using Gibson assembly. Mutations in the constructs were generated by standard mutagenic PCR using PfuUltra polymerase (QuikChange II; Agilent Technologies).

10.1128/mBio.03303-20.9 TABLE S1 Strains used in this study. Download Table S1, DOCX file, 0.01 MB. Copyright © 2021 Valastyan et al. 2021 Valastyan et al. https://creativecommons.org/licenses/by/4.0/ This content is distributed under the terms of the Creative Commons Attribution 4.0 International license.

10.1128/mBio.03303-20.10 TABLE S2 Plasmids used in this study. Download Table S2, DOCX file, 0.01 MB. Copyright © 2021 Valastyan et al. 2021 Valastyan et al. https://creativecommons.org/licenses/by/4.0/ This content is distributed under the terms of the Creative Commons Attribution 4.0 International license.

### Yeast, mammalian, and Z. rouxii AI-2 mimic production.

S. cerevisiae cells were grown overnight in YPD or SD-ura (as needed for plasmid maintenance) with shaking at 30°C. The cells were pelleted by centrifugation for 10 min at 4,000 rpm. The pellet was washed twice with sterile water, followed by centrifugation as above. The cells were resuspended in water and incubated for 24 h at 30°C with shaking. For making yeast AI-2 mimic for molecule identification, the cells were resuspended at OD_600_ of 100 and incubated overnight at 30°C. When making yeast AI-2 mimic for all other assays, unless otherwise stated, the cells were resuspended at an OD_600_ of 10 and incubated overnight at 30°C. The cells were removed by centrifugation as above, and the resulting cell-free fluids were filtered through 0.22-μm polyethersulfone (PES) membranes (Millipore Sigma). Such preparations were used as the sources of yeast AI-2 mimic for activity assays and for further purification. The mammalian AI-2 mimic produced by Caco-2 cells was prepared as described previously (13). *CFF1* was replaced with a KanMX cassette in Z. rouxii as previously described (77). Z. rouxii cell-free culture fluids were generated in water, as described above for S. cerevisiae.

### V. harveyi TL-25/TL-26 reporter assays.

Bioluminescence reporter assays using V. harveyi strains TL-25 and TL-26 were performed as previously reported (13, 78). Briefly, vibrio strains were grown overnight at 30°C in LM medium with shaking. V. harveyi cultures were diluted 1:1,000 in AB medium containing 100 μM boric acid. In all cases, cultures were aliquoted into wells of black-sided, clear-bottom 96-well plates (Corning). DPD, AI-1, MHF, or AI-2 mimic preparation was added at the indicated amounts, and the mixtures were serial diluted. Following incubation at 30°C with shaking for 6 h, bioluminescence and cell density (OD_600_) were measured using an Envision plate reader (PerkinElmer). Data are presented as relative light units (RLUs), which are bioluminescence per OD_600_. “Max AI-2” shown in figures indicates bioluminescence output following addition of 125 nM AI-2 (i.e., *S*-THMF-borate). “Max AI-1” depicted in figures indicates bioluminescence output following addition of 500 nM AI-1 (i.e., 3-hydroxyl C4-homoserine lactone).

### Yeast growth curve and survival assays.

S. cerevisiae strains were grown overnight at 30°C in YPD medium with shaking to saturation. The cultures were diluted to OD_600_ of 0.1 in a black-sided, clear-bottom 96-well plate. Cells were incubated at 30°C with shaking, and time points were taken every 20 min. Growth was measured in a Synergy plate reader (BioTek). For survival analyses, S. cerevisiae strains were diluted 1:100,000 and plated onto YPD agar plates. Colonies were allowed to grow for 48 h and then counted manually.

### Screen for S. cerevisiae genes required for MHF production.

The S. cerevisiae Yeast Knockout (YKO) Collection (Dharmacon) is a 96-well plate arrayed library containing about 5,000 S. cerevisiae strains with single gene deletions spanning the yeast genome (37–39). After thawing, 5 μl of each strain in the library was transferred into 96-well plates containing 150 μl YPD+G418 sulfate, and the plates were incubated at 30°C with shaking overnight. A total of 10 μl of each strain was diluted into 150-μl fresh YPD+G418 sulfate medium and allowed to grow for 5 h at 30°C with shaking. The cells were pelleted at 4,000 rpm for 10 min and then resuspended in water. The wash and centrifugation steps were repeated two more times. The cells were pelleted at 4,000 rpm for a final 10 min and resuspended in AB medium that had been supplemented with 100 μM boric acid. The initial OD_600_ of each culture was measured to identify any yeast mutants that had grown poorly. Yeast deletion strains that exhibited slowed growth were eliminated from analysis. The plates were incubated overnight at 30°C with shaking. The following morning, the plates were subjected to centrifugation at 4,000 rpm for 10 min. A total of 75 μl of culture fluid from each well was combined with 75 μl of fresh AB medium that had been inoculated with a 1:1,000 dilution of an overnight culture of the V. harveyi TL-26 reporter strain, and the mixtures were placed into fresh microtiter plates. The wells were supplemented with 100 μM boric acid (final concentration). Following 8 h of incubation at 30°C with aeration, bioluminescence and OD_600_ were measured (Envision Plate Reader). Normalized RLUs were calculated by dividing the bioluminescence by the OD_600_ and then dividing by the average RLUs from all plates from which measurements were made on a single day. Mutants from the screen that appeared to be defective in production of activity were retested individually, as above. Mutants of interest were reconstructed using the standard KanMX deletion method (38, 74) and again examined for the ability to produce activity.

### Cff1p alignment and phylogenetic tree production.

Potential Cff1p homologs were identified using BLASTp (50) and searching against S. cerevisiae Cff1p with an E value cutoff of 1e-5. This analysis returned 1,975 sequences. The list of sequences was culled to remove duplicate species resulting in 744 sequences. Rough phylogenetic analyses demonstrated that sequences from species within a genus clustered; thus, the list of sequences was further trimmed to include only the top hit in each genus, delivering 367 total sequences. These sequences were aligned using Clustal Omega (79) in SnapGene software (GSL Biotech). The phylogenetic tree in Fig. S7 was constructed in MEGA-X using the maximum likelihood method and Jones-Taylor-Thornton (JTT) matrix-based model with 500 bootstrap replications as described previously (80). The alignment was visualized using ggmsa in R, pruning the ends of the alignment to the first and last amino acids of S. cerevisiae Cff1p.

### Yeast AI-2 mimic purification and identification.

Approximately 250 ml of concentrated crude yeast AI-2 mimic preparation was filtered through a 0.45-μm polyvinylidene difluoride (PVDF) filter (Millipore Sigma) and separated on a 2- by 25-cm Luna C_18_ column (Phenomenex) using a mobile phase consisting of 5% water in methanol at a flow rate of 10 ml/min. The component of interest was eluted in 8 min in approximately 6 ml of mobile phase. The fraction was dried by rotary evaporation to remove methanol. To enable further concentration, the aqueous solution was saturated with sodium chloride and extracted with dichloromethane. The dichloromethane layer containing the product of interest was dried, and the sample was refrigerated. Fifty collections were processed in this manner. The products were combined and dissolved in 5 μl of deuterated water to a concentration of 0.003 mg/ml (determined subsequently by HPLC analysis using a standard curve). A “background” sample was generated by collecting the same volume of mobile phase in an area containing no UV-visible eluting components. Both samples were analyzed by ^1^H and ^13^C NMR spectroscopy, and although the data were inconclusive, several key signals were detected in the active sample that were not present in and not obscured by the background sample. For example, in the ^13^C NMR, signals at δ 194, 172, and 134 ppm were observed reproducibly. These data combined with mass spectrometry data led to a collection of possible candidates for the active component (Fig. S2A). The structure was confirmed by GC-MS analysis, including matching of the fragmentation pattern of the active component against a database of known structures. Analysis was carried out on an RTX-1ms (25 μm) 30-m column with 0.32-mm internal diameter (ID) (Restek). The samples were incubated at 40°C for 2 min and then subjected to a temperature ramp up of 10°C/min to 300°C, followed by a 5-min hold at 300°C. Mammalian AI-2 mimic activity was separated on a 25- by 0.46-cm Synergi 10-μm Polar-RP column using a mobile phase of 5% water in methanol at a flow rate of 0.5 ml/min.

### Protein gels.

To assess the levels of Cff1p, the Cff1p and Cff homologs, and the Cff1p and Cff variants, yeast cells producing the protein of interest were grown to exponential phase in SD-Ura. In each case, cells equivalent to 10 OD_600_ units were pelleted for 10 min at 4,000 rpm and resuspended in 50 μl yeast protein extraction reagent (Y-PER; Thermo Fisher Scientific) supplemented with 1× Halt protease inhibitor cocktail (Thermo Fisher Scientific). These mixtures were incubated at room temperature for 20 min with agitation and then subjected to centrifugation at 13,000 rpm in a tabletop centrifuge. The supernatants were collected and incubated with 1 μM HaloTag TMR ligand (Promega) at room temperature for 20 min. Next, 4× Laemmli sample buffer was added, and the mixtures were incubated at 70°C for 10 min. Samples were loaded onto 4% to 20% gradient mini-protean precast gels (Bio-Rad). Following electrophoresis, proteins were visualized using the Cy3 filter set on an ImageQuant LAS 4000 instrument. To assess total protein, gels were stained with Coomassie brilliant blue R-250 staining solution (Bio-Rad) and visualized using an ImageQuant LAS 4000 instrument.

### Data availability.

All data and materials published in the manuscript are available upon request.

## References

[1] Papenfort K, Bassler BL. 2016. Quorum sensing signal-response systems in Gram-negative bacteria. Nat Rev Microbiol 14:576–588. doi:10.1038/nrmicro.2016.89. 27510864 PMC5056591

[2] Eickhoff MJ, Bassler BL. 2018. SnapShot: bacterial quorum sensing. Cell 174:1328–1328.e1. doi:10.1016/j.cell.2018.08.003. 30142348

[3] Mukherjee S, Bassler BL. 2019. Bacterial quorum sensing in complex and dynamically changing environments. Nat Rev Microbiol 17:371–382. doi:10.1038/s41579-019-0186-5. 30944413 PMC6615036

[4] Bassler BL, Wright M, Showalter RE, Silverman MR. 1993. Intercellular signalling in Vibrio harveyi: sequence and function of genes regulating expression of luminescence. Mol Microbiol 9:773–786. doi:10.1111/j.1365-2958.1993.tb01737.x. 8231809

[5] Bassler BL, Wright M, Silverman MR. 1994. Multiple signalling systems controlling expression of luminescence in Vibrio harveyi: sequence and function of genes encoding a second sensory pathway. Mol Microbiol 13:273–286. doi:10.1111/j.1365-2958.1994.tb00422.x. 7984107

[6] Schauder S, Shokat K, Surette MG, Bassler BL. 2001. The LuxS family of bacterial autoinducers: biosynthesis of a novel quorum-sensing signal molecule. Mol Microbiol 41:463–476. doi:10.1046/j.1365-2958.2001.02532.x. 11489131

[7] Henke JM, Bassler BL. 2004. Three parallel quorum-sensing systems regulate gene expression in Vibrio harveyi. J Bacteriol 186:6902–6914. doi:10.1128/JB.186.20.6902-6914.2004. 15466044 PMC522208

[8] Chen X, Schauder S, Potier N, Dorsselaer AV, Pelczer I, Bassler BL, Hughson FM. 2002. Structural identification of a bacterial quorum-sensing signal containing boron. Nature 415:545–549. doi:10.1038/415545a. 11823863

[9] Neiditch MB, Federle MJ, Miller ST, Bassler BL, Hughson FM. 2005. Regulation of LuxPQ receptor activity by the quorum-sensing signal autoinducer-2. Mol Cell 18:507–518. doi:10.1016/j.molcel.2005.04.020. 15916958

[10] Federle MJ, Bassler BL. 2003. Interspecies communication in bacteria. J Clin Invest 112:1291–1299. doi:10.1172/JCI20195. 14597753 PMC228483

[11] Xavier KB, Bassler BL. 2005. Interference with AI-2-mediated bacterial cell–cell communication. Nature 437:750–753. doi:10.1038/nature03960. 16193054 PMC1388276

[12] Surette MG, Miller MB, Bassler BL. 1999. Quorum sensing in Escherichia coli, Salmonella typhimurium, and Vibrio harveyi: a new family of genes responsible for autoinducer production. Proc Natl Acad Sci U S A 96:1639–1644. doi:10.1073/pnas.96.4.1639. 9990077 PMC15544

[13] Ismail AS, Valastyan JS, Bassler BL. 2016. A host-produced autoinducer-2 mimic activates bacterial quorum sensing. Cell Host Microbe 19:470–480. doi:10.1016/j.chom.2016.02.020. 26996306 PMC4869860

[14] Miller ST, Xavier KB, Campagna SR, Taga ME, Semmelhack MF, Bassler BL, Hughson FM. 2004. Salmonella Typhimurium recognizes a chemically distinct form of the bacterial quorum-sensing signal AI-2. Mol Cell 15:677–687. doi:10.1016/j.molcel.2004.07.020. 15350213

[15] Zhang L, Li S, Liu X, Wang Z, Jiang M, Wang R, Xie L, Liu Q, Xie X, Shang D, Li M, Wei Z, Wang Y, Fan C, Luo Z-Q, Shen X. 2020. Sensing of autoinducer-2 by functionally distinct receptors in prokaryotes. Nat Commun 11:5371. doi:10.1038/s41467-020-19243-5. 33097715 PMC7584622

[16] Chen H, Fink GR. 2006. Feedback control of morphogenesis in fungi by aromatic alcohols. Genes Dev 20:1150–1161. doi:10.1101/gad.1411806. 16618799 PMC1472474

[17] Chen H, Fujita M, Feng Q, Clardy J, Fink GR. 2004. Tyrosol is a quorum-sensing molecule in Candida albicans. Proc Natl Acad Sci U S A 101:5048–5052. doi:10.1073/pnas.0401416101. 15051880 PMC387371

[18] Oh K-B, Miyazawa H, Naito T, Matsuoka H. 2001. Purification and characterization of an autoregulatory substance capable of regulating the morphological transition in Candida albicans. Proc Natl Acad Sci U S A 98:4664–4668. doi:10.1073/pnas.071404698. 11274356 PMC31891

[19] Hornby JM, Jensen EC, Lisec AD, Tasto JJ, Jahnke B, Shoemaker R, Dussault P, Nickerson KW. 2001. Quorum sensing in the dimorphic fungus Candida albicans is mediated by farnesol. Appl Environ Microbiol 67:2982–2992. doi:10.1128/AEM.67.7.2982-2992.2001. 11425711 PMC92970

[20] Padder SA, Prasad R, Shah AH. 2018. Quorum sensing: a less known mode of communication among fungi. Microbiol Res 210:51–58. doi:10.1016/j.micres.2018.03.007. 29625658

[21] Cugini C, Calfee MW, Farrow JM, Morales DK, Pesci EC, Hogan DA. 2007. Farnesol, a common sesquiterpene, inhibits PQS production in Pseudomonas aeruginosa. Mol Microbiol 65:896–906. doi:10.1111/j.1365-2958.2007.05840.x. 17640272

[22] De Sordi L, Mühlschlegel FA. 2009. Quorum sensing and fungal-bacterial interactions in Candida albicans: a communicative network regulating microbial coexistence and virulence. FEMS Yeast Res 9:990–999. doi:10.1111/j.1567-1364.2009.00573.x. 19845041

[23] Polke M, Leonhardt I, Kurzai O, Jacobsen ID. 2018. Farnesol signalling in Candida albicans—more than just communication. Crit Rev Microbiol 44:230–243. doi:10.1080/1040841X.2017.1337711. 28609183

[24] Leonhardt I, Spielberg S, Weber M, Albrecht-Eckardt D, Bläss M, Claus R, Barz D, Scherlach K, Hertweck C, Löffler J, Hünniger K, Kurzai O. 2015. The fungal quorum-sensing molecule farnesol activates innate immune cells but suppresses cellular adaptive immunity. mBio 6:e00143-15. doi:10.1128/mBio.00143-15. 25784697 PMC4453522

[25] Hargarten JC, Moore TC, Petro TM, Nickerson KW, Atkin AL. 2015. Candida albicans quorum sensing molecules stimulate mouse macrophage migration. Infect Immun 83:3857–3864. doi:10.1128/IAI.00886-15. 26195556 PMC4567634

[26] Zhou C-Z, Meyer P, Quevillon-Cheruel S, Li De La Sierra-Gallay I, Collinet B, Graille M, Blondeau K, François J-M, Leulliot N, Sorel I, Poupon A, Janin J, Van Tilbeurgh H. 2005. Crystal structure of the YML079w protein from Saccharomyces cerevisiae reveals a new sequence family of the jelly-roll fold. Protein Sci 14:209–215. doi:10.1110/ps.041121305. 15608122 PMC2253319

[27] Kachroo AH, Laurent JM, Yellman CM, Meyer AG, Wilke CO, Marcotte EM. 2015. Systematic humanization of yeast genes reveals conserved functions and genetic modularity. Science 348:921–925. doi:10.1126/science.aaa0769. 25999509 PMC4718922

[28] Duina AA, Miller ME, Keeney JB. 2014. Budding yeast for budding geneticists: a primer on the Saccharomyces cerevisiae model system. Genetics 197:33–48. doi:10.1534/genetics.114.163188. 24807111 PMC4012490

[29] Long T, Tu KC, Wang Y, Mehta P, Ong NP, Bassler BL, Wingreen NS. 2009. Quantifying the integration of quorum-sensing signals with single-cell resolution. PLoS Biol 7:e68. doi:10.1371/journal.pbio.1000068. 19320539 PMC2661960

[30] Lewis JA, Elkon IM, McGee MA, Higbee AJ, Gasch AP. 2010. Exploiting natural variation in Saccharomyces cerevisiae to identify genes for increased ethanol resistance. Genetics 186:1197–1205. doi:10.1534/genetics.110.121871. 20855568 PMC2998304

[31] Stuecker TN, Scholes AN, Lewis JA. 2018. Linkage mapping of yeast cross protection connects gene expression variation to a higher-order organismal trait. PLoS Genet 14:e1007335. doi:10.1371/journal.pgen.1007335. 29649251 PMC5978988

[32] Cao JG, Meighen EA. 1989. Purification and structural identification of an autoinducer for the luminescence system of Vibrio harveyi. J Biol Chem 264:21670–21676. doi:10.1016/S0021-9258(20)88238-6. 2600086

[33] Huang X, Duddy OP, Silpe JE, Paczkowski JE, Cong J, Henke BR, Bassler BL. 2020. Mechanism underlying autoinducer recognition in the Vibrio cholerae DPO-VqmA quorum-sensing pathway. J Biol Chem 295:2916–2931. doi:10.1074/jbc.RA119.012104. 31964715 PMC7062168

[34] Ke X, Miller LC, Bassler BL. 2015. Determinants governing ligand specificity of the Vibrio harveyi LuxN quorum-sensing receptor. Mol Microbiol 95:127–142. doi:10.1111/mmi.12852. 25367076 PMC4275348

[35] McCready AR, Paczkowski JE, Henke BR, Bassler BL. 2019. Structural determinants driving homoserine lactone ligand selection in the Pseudomonas aeruginosa LasR quorum-sensing receptor. Proc Natl Acad Sci U S A 116:245–254. doi:10.1073/pnas.1817239116. 30559209 PMC6320529

[36] Boursier ME, Moore JD, Heitman KM, Shepardson-Fungairino SP, Combs JB, Koenig LC, Shin D, Brown EC, Nagarajan R, Blackwell HE. 2018. Structure-function analyses of the N-butanoyl l-homoserine lactone quorum-sensing signal define features critical to activity in RhlR. ACS Chem Biol 13:2655–2662. doi:10.1021/acschembio.8b00577. 30114353 PMC6200399

[37] Winzeler EA, Shoemaker DD, Astromoff A, Liang H, Anderson K, Andre B, Bangham R, Benito R, Boeke JD, Bussey H, Chu AM, Connelly C, Davis K, Dietrich F, Dow SW, El Bakkoury M, Foury F, Friend SH, Gentalen E, Giaever G, Hegemann JH, Jones T, Laub M, Liao H, Liebundguth N, Lockhart DJ, Lucau-Danila A, Lussier M, M’Rabet N, Menard P, Mittmann M, Pai C, Rebischung C, Revuelta JL, Riles L, Roberts CJ, Ross-MacDonald P, Scherens B, Snyder M, Sookhai-Mahadeo S, Storms RK, Véronneau S, Voet M, Volckaert G, Ward TR, Wysocki R, Yen GS, Yu K, Zimmermann K, Philippsen P, Johnston M, . 1999. Functional characterization of the S. cerevisiae genome by gene deletion and parallel analysis. Science 285:901–906. doi:10.1126/science.285.5429.901. 10436161

[38] Wach A, Brachat A, Pöhlmann R, Philippsen P. 1994. New heterologous modules for classical or PCR-based gene disruptions in Saccharomyces cerevisiae. Yeast 10:1793–1808. doi:10.1002/yea.320101310. 7747518

[39] Giaever G, Chu AM, Ni L, Connelly C, Riles L, Véronneau S, Dow S, Lucau-Danila A, Anderson K, André B, Arkin AP, Astromoff A, El-Bakkoury M, Bangham R, Benito R, Brachat S, Campanaro S, Curtiss M, Davis K, Deutschbauer A, Entian K-D, Flaherty P, Foury F, Garfinkel DJ, Gerstein M, Gotte D, Güldener U, Hegemann JH, Hempel S, Herman Z, Jaramillo DF, Kelly DE, Kelly SL, Kötter P, LaBonte D, Lamb DC, Lan N, Liang H, Liao H, Liu L, Luo C, Lussier M, Mao R, Menard P, Ooi SL, Revuelta JL, Roberts CJ, Rose M, Ross-Macdonald P, Scherens B, Schimmack G, et al. 2002. Functional profiling of the Saccharomyces cerevisiae genome. Nature 418:387–391. doi:10.1038/nature00935. 12140549

[40] Planta RJ, Mager WH. 1998. The list of cytoplasmic ribosomal proteins of Saccharomyces cerevisiae. Yeast 14:471–477. doi:10.1002/(SICI)1097-0061(19980330)14:5<471::AID-YEA241>3.0.CO;2-U. 9559554

[41] Yoshikawa K, Tanaka T, Ida Y, Furusawa C, Hirasawa T, Shimizu H. 2011. Comprehensive phenotypic analysis of single-gene deletion and overexpression strains of Saccharomyces cerevisiae. Yeast 28:349–361. doi:10.1002/yea.1843. 21341307

[42] Hauck T, Landmann C, Brühlmann F, Schwab W. 2003. Formation of 5-methyl-4-hydroxy-3[2H]-furanone in cytosolic extracts obtained from Zygosaccharomyces rouxii. J Agric Food Chem 51:1410–1414. doi:10.1021/jf025948m. 12590490

[43] Knowles FC, Chanley JD, Pon NG. 1980. Spectral changes arising from the action of spinach chloroplast ribosephosphate isomerase on ribose 5-phosphate. Arch Biochem Biophys 202:106–115. doi:10.1016/0003-9861(80)90411-7. 7396530

[44] Hauck T, Hübner Y, Brühlmann F, Schwab W. 2003. Alternative pathway for the formation of 4,5-dihydroxy-2,3-pentanedione, the proposed precursor of 4-hydroxy-5-methyl-3(2H)-furanone as well as autoinducer-2, and its detection as natural constituent of tomato fruit. Biochim Biophys Acta 1623:109–119. doi:10.1016/j.bbagen.2003.08.002. 14572908

[45] Sasaki M, Nunomura N, Matsudo T. 1991. Biosynthesis of 4-hydroxy-2(or 5)-ethyl-5(or 2)-methyl-3(2H)-furanone by yeasts. J Agric Food Chem 39:934–938. doi:10.1021/jf00005a027.

[46] Hicks K, Harris D, Feather M, Loeppky R. 1974. Production of 4-hydroxy-5-methyl-3(2H)-furanone, a component of beef flavor, from a 1-amino-1-deoxy-d-fructuronic acid. J Agric Food Chem 22:724–725. doi:10.1021/jf60194a005.

[47] Hicks K, Feather MS. 1975. Studies on the mechanism of formation of 4-hydroxy-5-methyl-3(2H)-furanone, a component of beef flavor, from Amadori products. J Agric Food Chem 23:957–960. doi:10.1021/jf60201a005.

[48] Dunwell JM. 1998. Cupins: a new superfamily of functionally diverse proteins that include germins and plant storage proteins. Biotechnol Genet Eng Rev 15:1–32. doi:10.1080/02648725.1998.10647950. 9573603

[49] Tourneroche A, Lami R, Hubas C, Blanchet E, Vallet M, Escoubeyrou K, Paris A, Prado S. 2019. Bacterial-fungal interactions in the kelp endomicrobiota drive autoinducer-2 quorum sensing. Front Microbiol 10:1693. doi:10.3389/fmicb.2019.01693. 31417510 PMC6685064

[50] Altschul SF, Gish W, Miller W, Myers EW, Lipman DJ. 1990. Basic local alignment search tool. J Mol Biol 215:403–410. doi:10.1016/S0022-2836(05)80360-2. 2231712

[51] Mitchell AL, Attwood TK, Babbitt PC, Blum M, Bork P, Bridge A, Brown SD, Chang H-Y, El-Gebali S, Fraser MI, Gough J, Haft DR, Huang H, Letunic I, Lopez R, Luciani A, Madeira F, Marchler-Bauer A, Mi H, Natale DA, Necci M, Nuka G, Orengo C, Pandurangan AP, Paysan-Lafosse T, Pesseat S, Potter SC, Qureshi MA, Rawlings ND, Redaschi N, Richardson LJ, Rivoire C, Salazar GA, Sangrador-Vegas A, Sigrist CJA, Sillitoe I, Sutton GG, Thanki N, Thomas PD, Tosatto SCE, Yong S-Y, Finn RD. 2019. InterPro in 2019: improving coverage, classification and access to protein sequence annotations. Nucleic Acids Res 47:D351–D360. doi:10.1093/nar/gky1100. 30398656 PMC6323941

[52] Honkanen E, Pyysalo T, Hirvi T. 1980. The aroma of finnish wild raspberries, Rubus idaeus, L. Z Lebensm Unters Forch 171:180–182. doi:10.1007/BF01042646.

[53] Idstein H, Schreier P. 1985. Volatile constituents from guava (Psidium guajava, L.) fruit. J Agric Food Chem 33:138–143. doi:10.1021/jf00061a039.

[54] Farine J-P, Qvere J-LL, Duffy J, Semon E, Brossut R. 1993. 4-Hydroxy-5-methyl-3(2H)-furanone and 4-hydroxy-2,5-dimethyl-3(2H)-furanone, two components of the male sex pheromone of Eurycotis floridana (Walker) (Insecta, Blattidae, Polyzosteriinae. Biosci Biotechnol Biochem 57:2026–2030. doi:10.1271/bbb.57.2026.

[55] Li Q, Ren Y, Fu X. 2019. Inter-kingdom signaling between gut microbiota and their host. Cell Mol Life Sci 76:2383–2389. doi:10.1007/s00018-019-03076-7. 30911771 PMC11105296

[56] Fourie R, Ells R, Swart CW, Sebolai OM, Albertyn J, Pohl CH. 2016. Candida albicans and Pseudomonas aeruginosa interaction, with focus on the role of eicosanoids. Front Physiol 7:64. doi:10.3389/fphys.2016.00064. 26955357 PMC4767902

[57] Simon S, Schell U, Heuer N, Hager D, Albers MF, Matthias J, Fahrnbauer F, Trauner D, Eichinger L, Hedberg C, Hilbi H. 2015. Inter-kingdom signaling by the Legionella quorum sensing molecule LAI-1 modulates cell migration through an IQGAP1-Cdc42-ARHGEF9-dependent pathway. PLoS Pathog 11:e1005307. doi:10.1371/journal.ppat.1005307. 26633832 PMC4669118

[58] Silpe JE, Bassler BL. 2019. Phage-encoded LuxR-type receptors responsive to host-produced bacterial quorum-sensing autoinducers. mBio 10:e00638-19. doi:10.1128/mBio.00638-19. 30967469 PMC6456758

[59] Silpe JE, Bassler BL. 2019. A host-produced quorum-sensing autoinducer controls a phage lysis-lysogeny decision. Cell 176:268–280.e13. doi:10.1016/j.cell.2018.10.059. 30554875 PMC6329655

[60] Silpe JE, Bridges AA, Huang X, Coronado DR, Duddy OP, Bassler BL. 2020. Separating functions of the phage-encoded quorum-sensing-activated antirepressor Qtip. Cell Host Microbe 27:629–641.e4. doi:10.1016/j.chom.2020.01.024. 32101705 PMC7148176

[61] Pereira CS, Thompson JA, Xavier KB. 2013. AI-2-mediated signalling in bacteria. FEMS Microbiol Rev 37:156–181. doi:10.1111/j.1574-6976.2012.00345.x. 22712853

[62] Hayashida Y, Slaughter JC. 1997. Biosynthesis of flavour-active furanones by Saccharomyces cerevisiae during fermentation depends on the malt type used in medium preparation. Biotechnol Lett 19:429–431. doi:10.1023/A:1018335925239.

[63] Ho C-T, Li J, Kuo M-C. 1999. Flavor chemistry of selected condiments and spices used in Chinese foods, p 55–76. *In* Shahidi F, Ho C-T (ed), Flavor chemistry of ethnic foods. Springer US, Boston, MA.

[64] Luh BS. 1995. Industrial production of soy sauce. J Ind Microbiol 14:467–471. doi:10.1007/BF01573959.

[65] Tavender T, Halliday N, Hardie K, Winzer K. 2008. LuxS-independent formation of AI-2 from ribulose-5-phosphate. BMC Microbiol 8:98. doi:10.1186/1471-2180-8-98. 18564424 PMC2443158

[66] Giraud MF, Leonard GA, Field RA, Berlind C, Naismith JH. 2000. RmlC, the third enzyme of dTDP-L-rhamnose pathway, is a new class of epimerase. Nat Struct Biol 7:398–402. doi:10.1038/75178. 10802738

[67] Jouhten P, Ponomarova O, Gonzalez R, Patil KR. 2016. Saccharomyces cerevisiae metabolism in ecological context. FEMS Yeast Res 16:fow080. doi:10.1093/femsyr/fow080. 27634775 PMC5050001

[68] Rowan-Nash AD, Korry BJ, Mylonakis E, Belenky P. 2019. Cross-domain and viral interactions in the microbiome. Microbiol Mol Biol Rev 83:e00044-18. doi:10.1128/MMBR.00044-18. 30626617 PMC6383444

[69] De Sordi L, Lourenço M, Debarbieux L. 2019. The battle within: interactions of bacteriophages and bacteria in the gastrointestinal tract. Cell Host Microbe 25:210–218. doi:10.1016/j.chom.2019.01.018. 30763535

[70] Hansen MF, Svenningsen SL, Røder HL, Middelboe M, Burmølle M. 2019. Big impact of the tiny: bacteriophage-bacteria interactions in biofilms. Trends Microbiol 27:739–752. doi:10.1016/j.tim.2019.04.006. 31128928

[71] Romano JD, Kolter R. 2005. Pseudomonas-Saccharomyces interactions: influence of fungal metabolism on bacterial physiology and survival. J Bacteriol 187:940–948. doi:10.1128/JB.187.3.940-948.2005. 15659672 PMC545695

[72] Ponomarova O, Gabrielli N, Sévin DC, Mülleder M, Zirngibl K, Bulyha K, Andrejev S, Kafkia E, Typas A, Sauer U, Ralser M, Patil KR. 2017. Yeast creates a niche for symbiotic lactic acid bacteria through nitrogen overflow. Cell Syst 5:345–357.e6. doi:10.1016/j.cels.2017.09.002. 28964698 PMC5660601

[73] Smith MG, Des Etages SG, Snyder M. 2004. Microbial synergy via an ethanol-triggered pathway. Mol Cell Biol 24:3874–3884. doi:10.1128/mcb.24.9.3874-3884.2004. 15082781 PMC387754

[74] Baudin A, Ozier-Kalogeropoulos O, Denouel A, Lacroute F, Cullin C. 1993. A simple and efficient method for direct gene deletion in Saccharomyces cerevisiae. Nucleic Acids Res 21:3329–3330. doi:10.1093/nar/21.14.3329. 8341614 PMC309783

[75] Los GV, Encell LP, McDougall MG, Hartzell DD, Karassina N, Zimprich C, Wood MG, Learish R, Ohana RF, Urh M, Simpson D, Mendez J, Zimmerman K, Otto P, Vidugiris G, Zhu J, Darzins A, Klaubert DH, Bulleit RF, Wood KV. 2008. HaloTag: a novel protein labeling technology for cell imaging and protein analysis. ACS Chem Biol 3:373–382. doi:10.1021/cb800025k. 18533659

[76] Sikorski RS, Hieter P. 1989. A system of shuttle vectors and yeast host strains designed for efficient manipulation of DNA in Saccharomyces cerevisiae. Genetics 122:19–27. doi:10.1093/genetics/122.1.19. 2659436 PMC1203683

[77] Watanabe J, Uehara K, Mogi Y, Suzuki K, Watanabe T, Yamazaki T. 2010. Improved transformation of the halo-tolerant yeast Zygosaccharomyces rouxii by electroporation. Biosci Biotechnol Biochem 74:1092–1094. doi:10.1271/bbb.90865. 20460697

[78] Bridges AA, Bassler BL. 2019. The intragenus and interspecies quorum-sensing autoinducers exert distinct control over Vibrio cholerae biofilm formation and dispersal. PLoS Biol 17:e3000429. doi:10.1371/journal.pbio.3000429. 31710602 PMC6872173

[79] Sievers F, Wilm A, Dineen D, Gibson TJ, Karplus K, Li W, Lopez R, McWilliam H, Remmert M, Söding J, Thompson JD, Higgins DG. 2011. Fast, scalable generation of high-quality protein multiple sequence alignments using Clustal Omega. Mol Syst Biol 7:539. doi:10.1038/msb.2011.75. 21988835 PMC3261699

[80] Kumar S, Stecher G, Li M, Knyaz C, Tamura K. 2018. MEGA X: molecular evolutionary genetics analysis across computing platforms. Mol Biol Evol 35:1547–1549. doi:10.1093/molbev/msy096. 29722887 PMC5967553

