# Supplementary figures and images for: Saccharomyces cerevisiae Requires CFF1 To Produce 4-Hydroxy-5-Methylfuran-3(2H)-One, a Mimic of the Bacterial Quorum-Sensing Autoinducer AI-2
Source: mBio. 2021 Mar 9;12(2):e03303-20. doi: 10.1128/mBio.03303-20 (PMC8092285; doi:10.1128/mBio.03303-20)

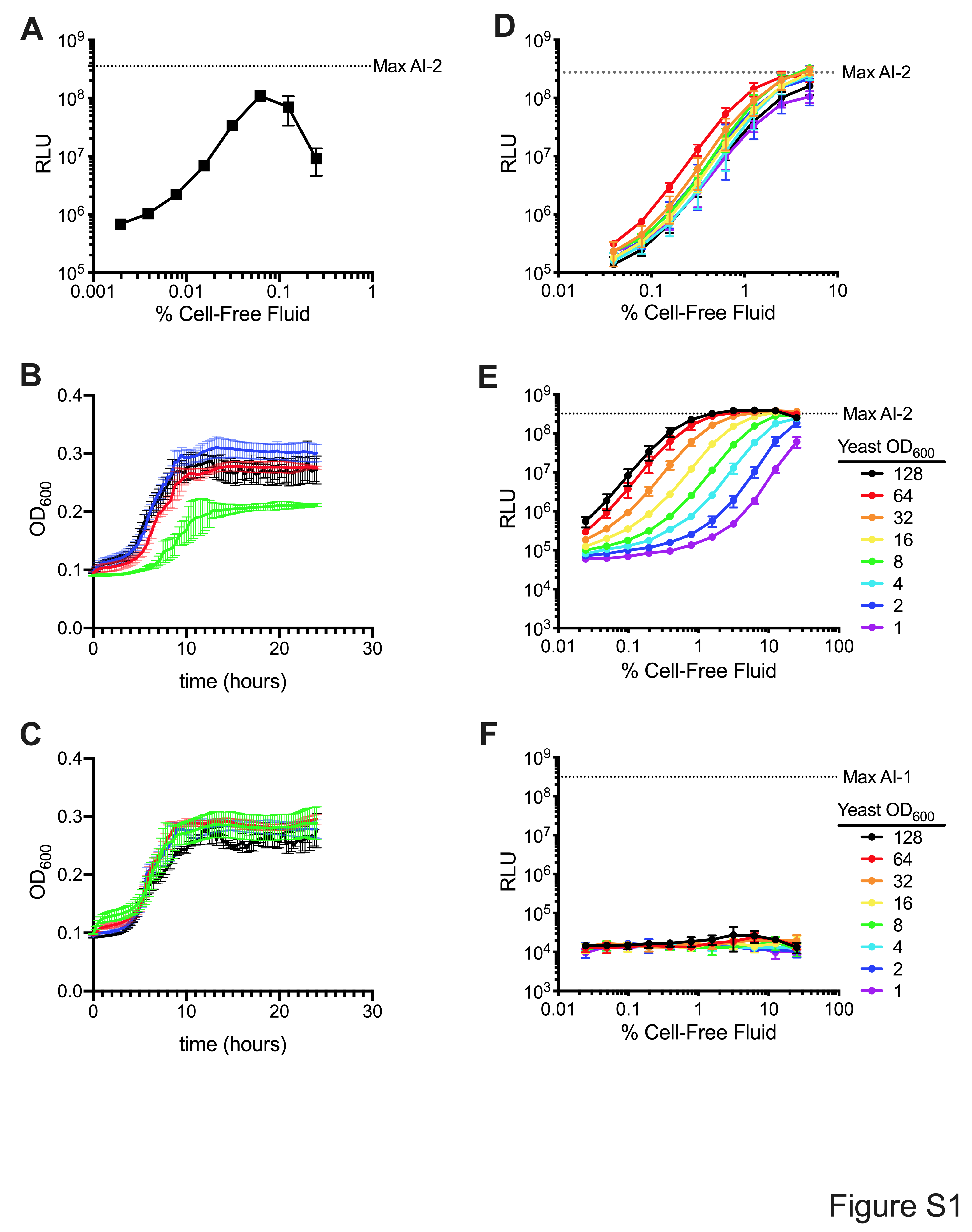

Supplement: FIG S1 [file mBio.03303-20-sf001.tif]

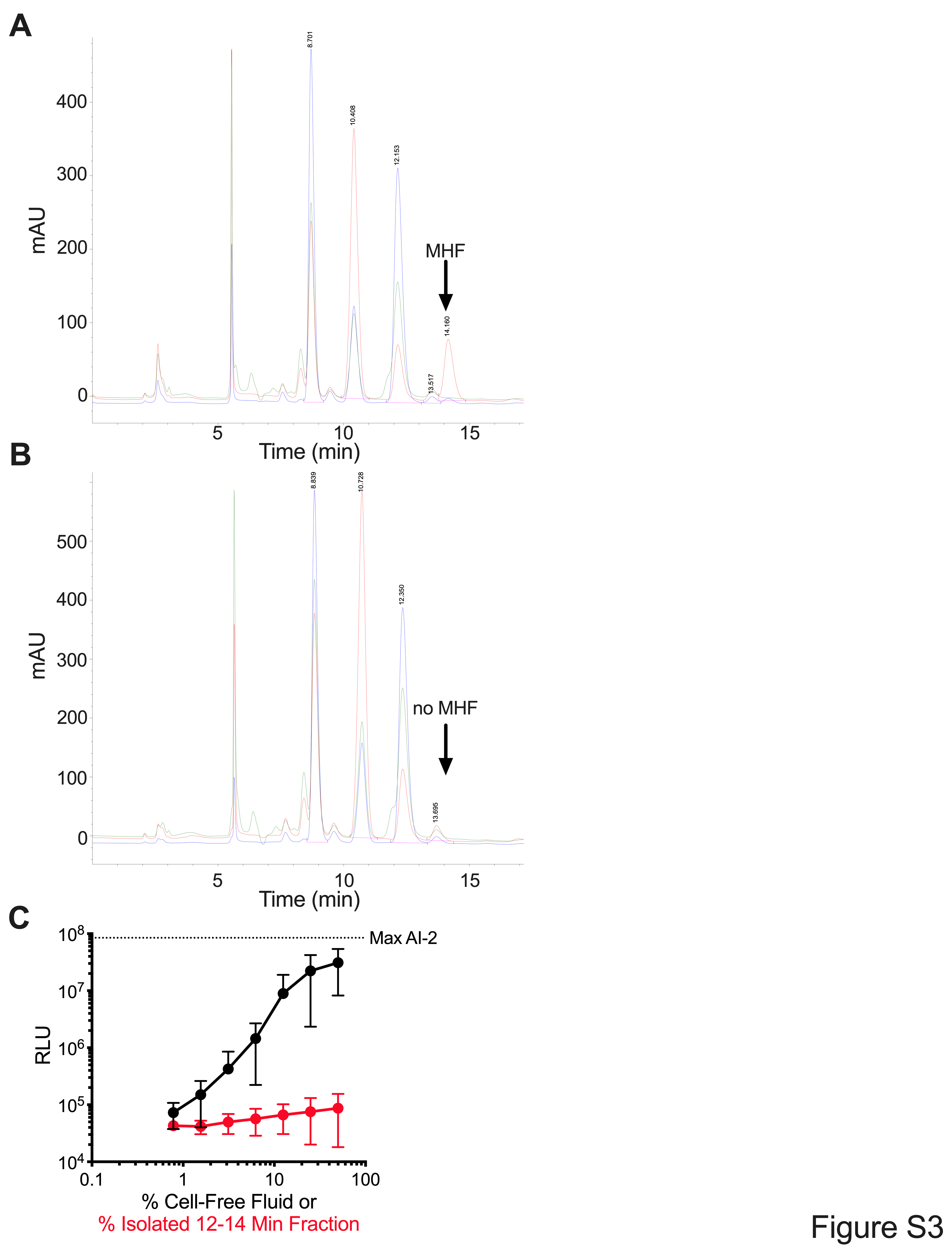

Supplement: FIG S3 [file mBio.03303-20-sf003.tif]

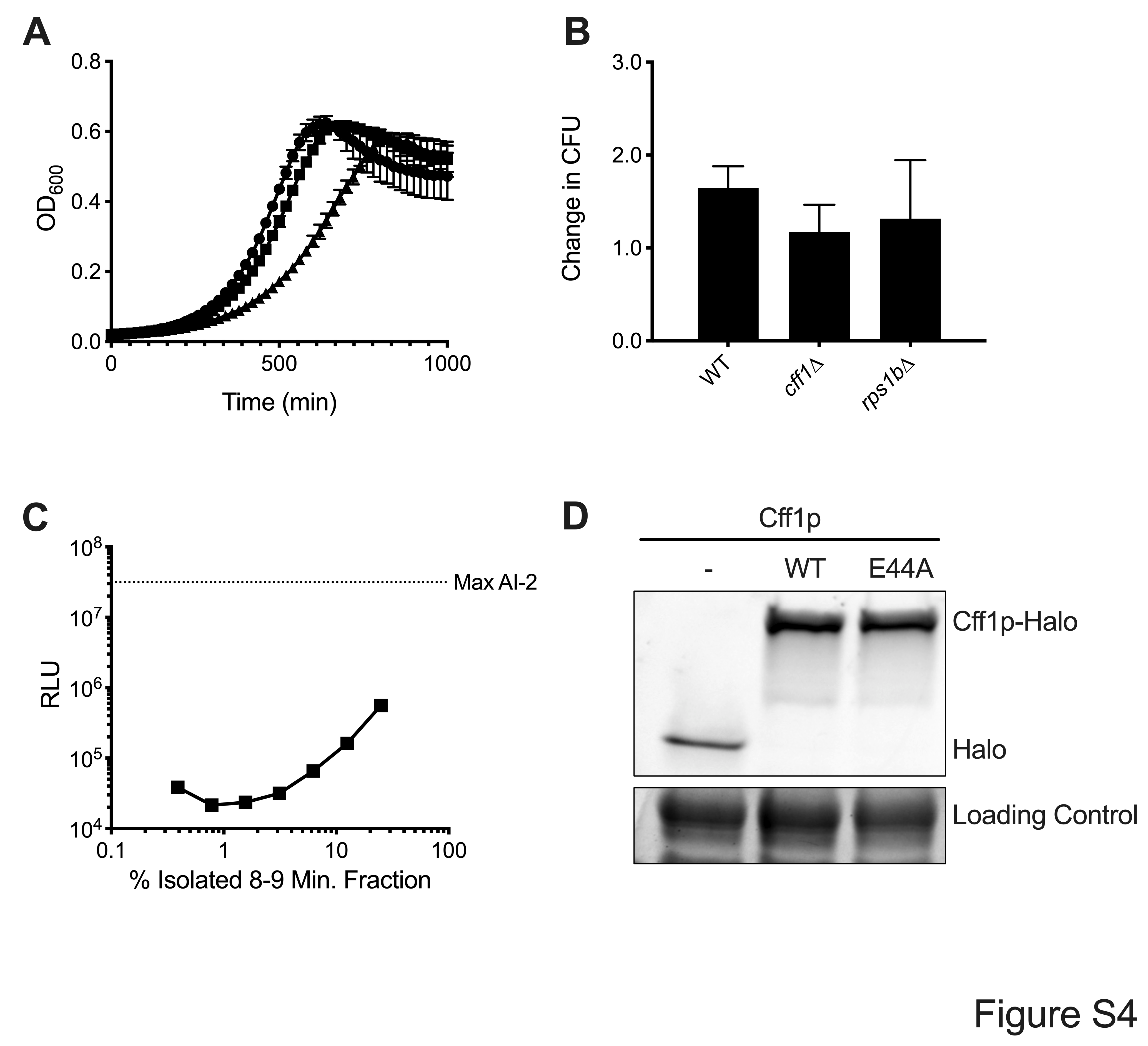

Supplement: FIG S4 [file mBio.03303-20-sf004.tif]

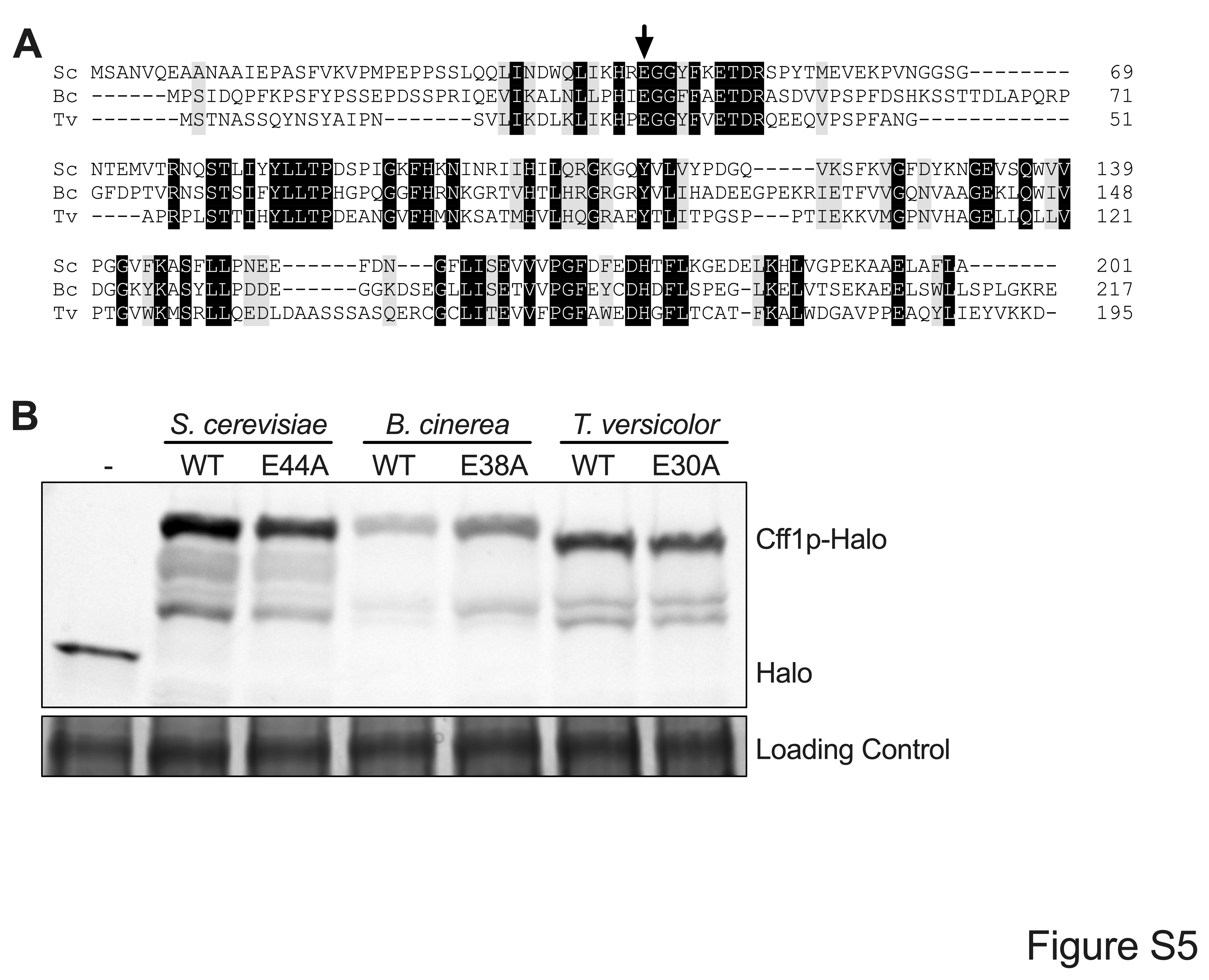

Supplement: FIG S5 [file mBio.03303-20-sf005.tif]

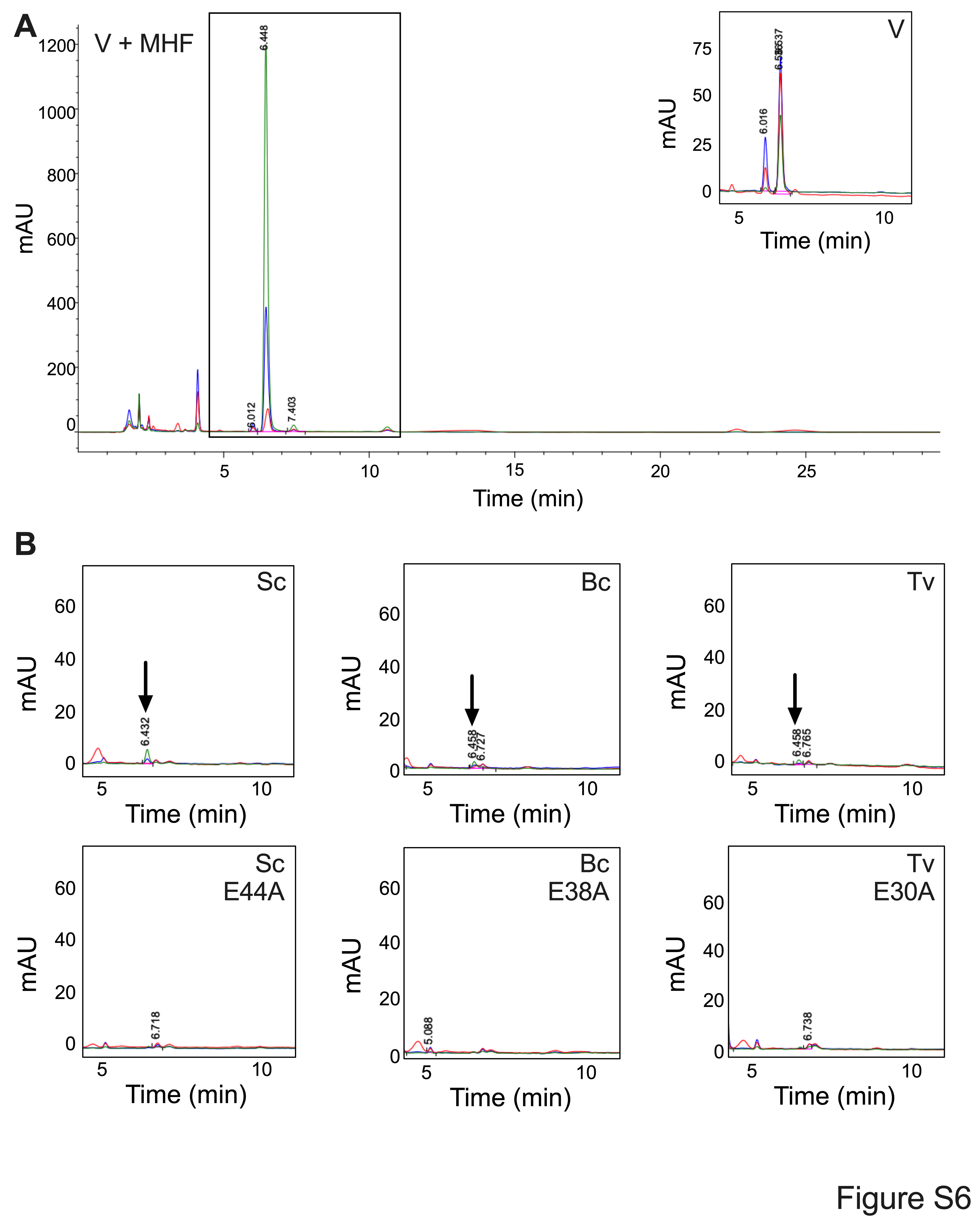

Supplement: FIG S6 [file mBio.03303-20-sf006.tif]

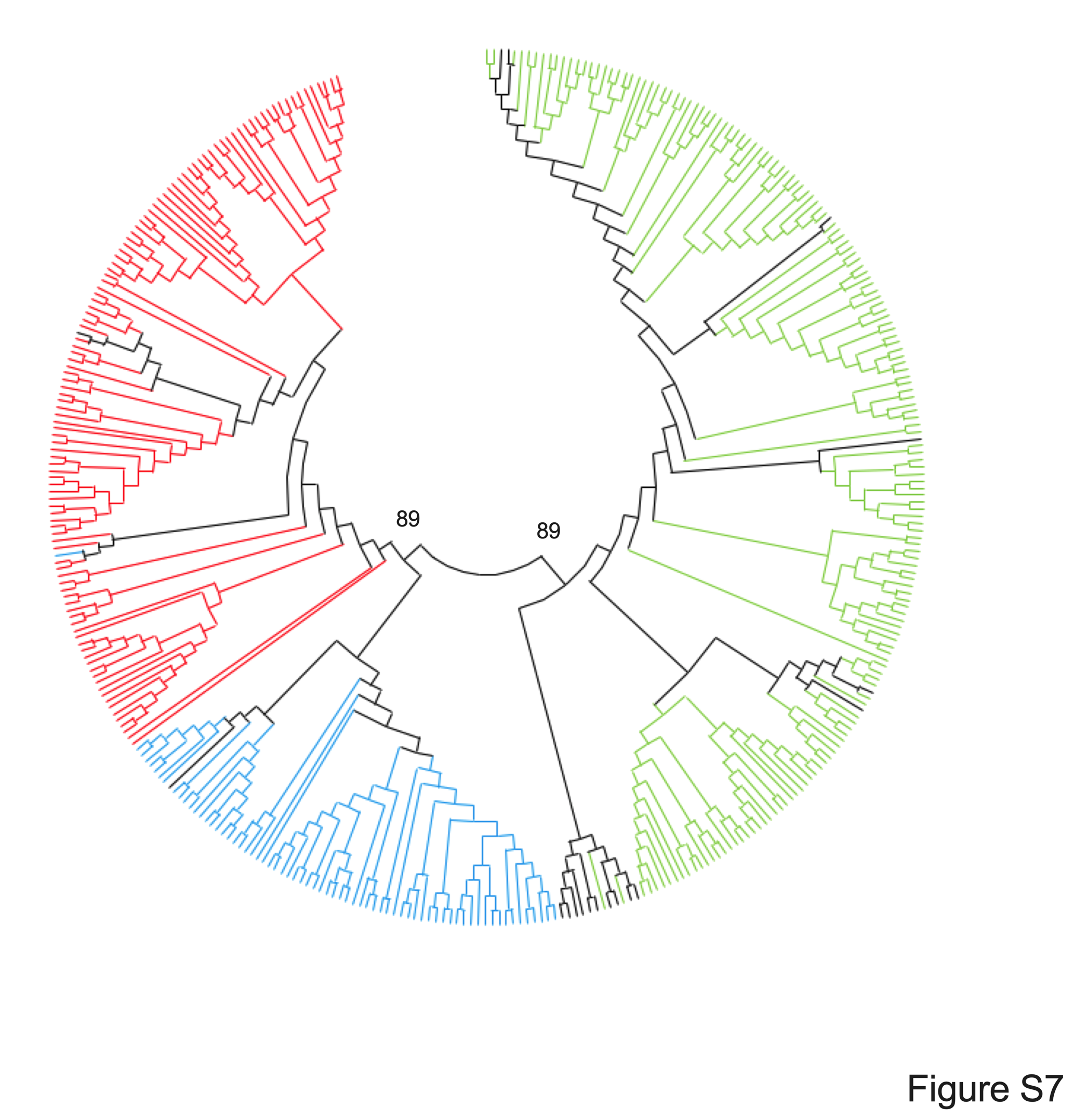

Supplement: FIG S7 [file mBio.03303-20-sf007.tif]

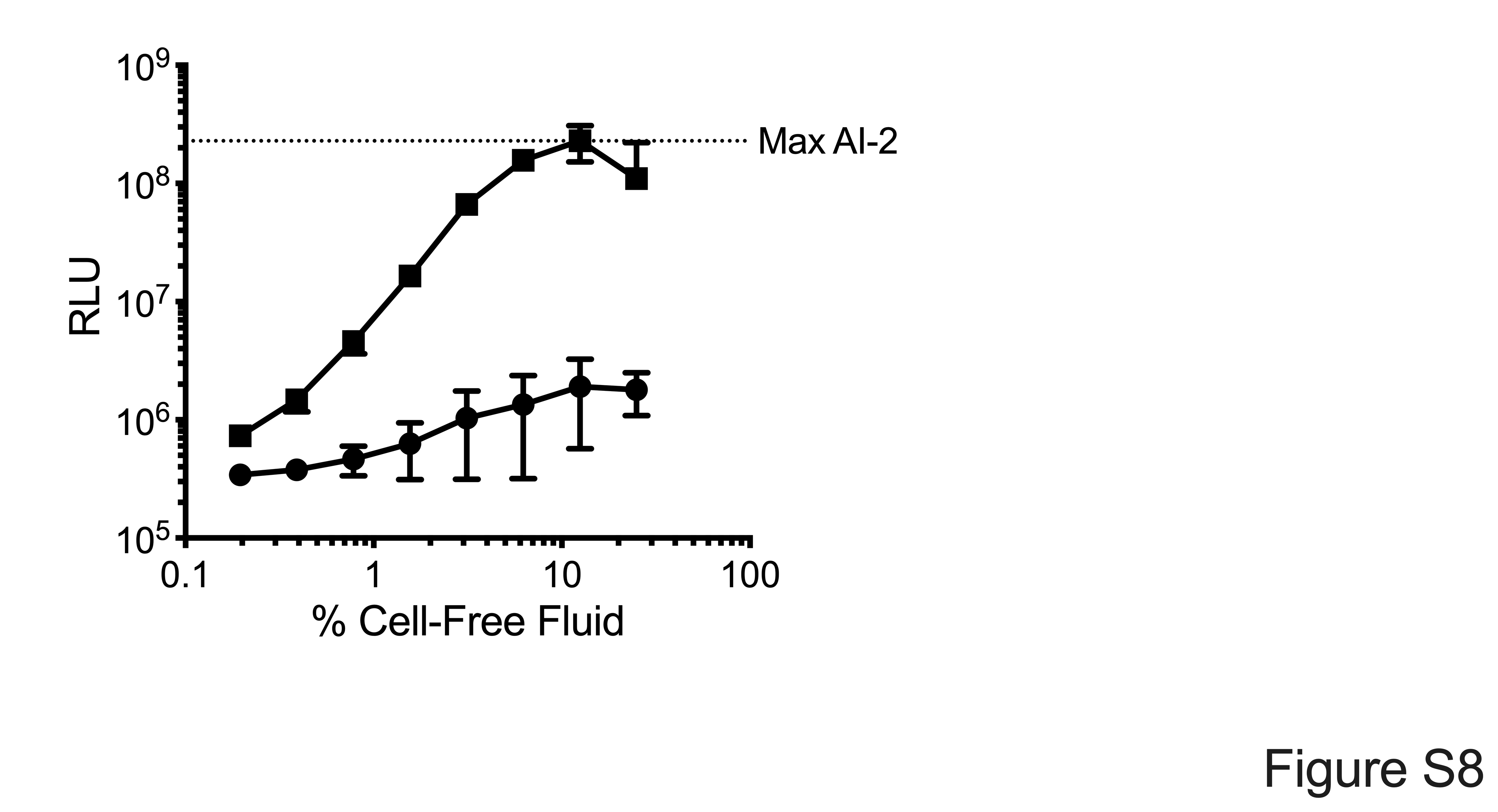

Supplement: FIG S8 [file mBio.03303-20-sf008.tif]
