# Supplementary material for: Saccharomyces cerevisiae Requires CFF1 To Produce 4-Hydroxy-5-Methylfuran-3(2H)-One, a Mimic of the Bacterial Quorum-Sensing Autoinducer AI-2
Source: mBio. 2021 Mar 9;12(2):e03303-20. doi: 10.1128/mBio.03303-20 (PMC8092285; doi:10.1128/mBio.03303-20)
Supplement: FIG S2 [file mBio.03303-20-sf002.pdf]

A

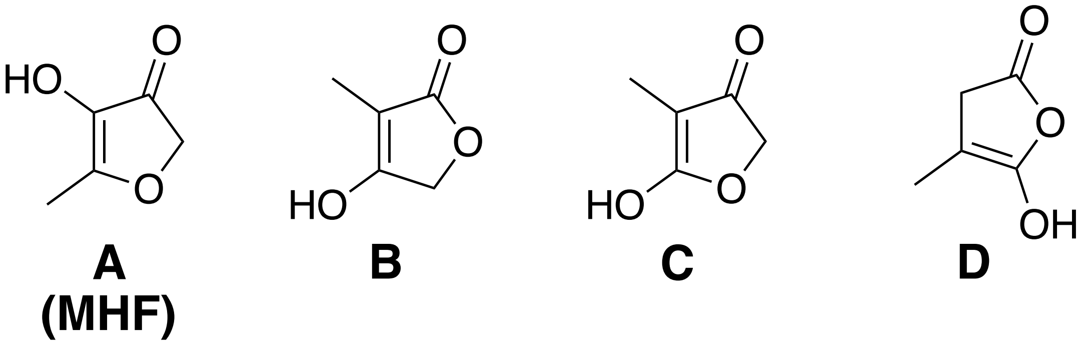

HO
O
O
A
(MHF)
O
O
HO
B
O
O
HO
C
O
O
OH
D

B

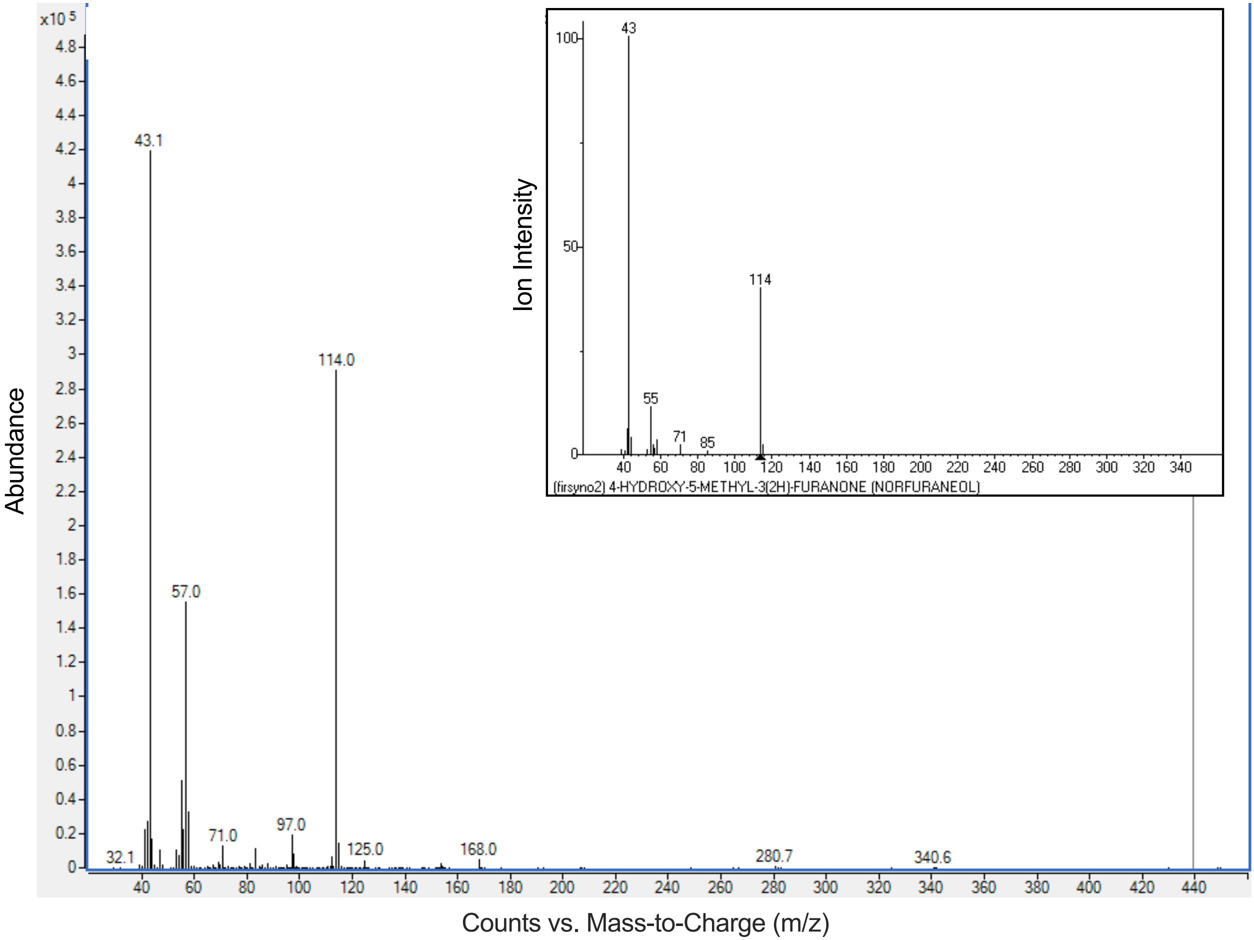

x10 5
Abundance
43.1
57.0
114.0
32.1
71.0
97.0
125.0
168.0
280.7
340.6
Counts vs. Mass-to-Charge (m/z)
Ion Intensity
43
114
55
71
85
(firsyno2) 4-HYDROXY-5-METHYL-3(2H)-FURANONE (NORFURANEOL)

Figure S2

C

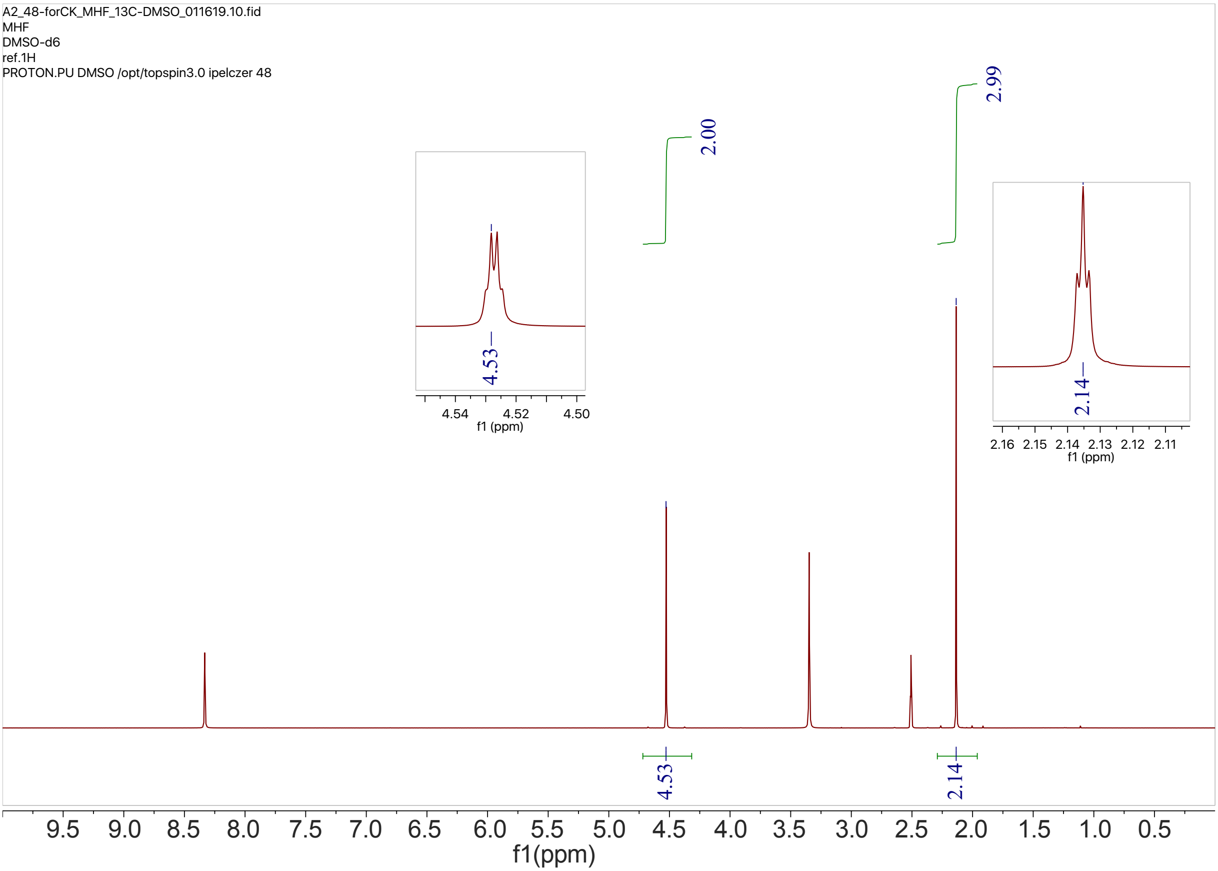

D

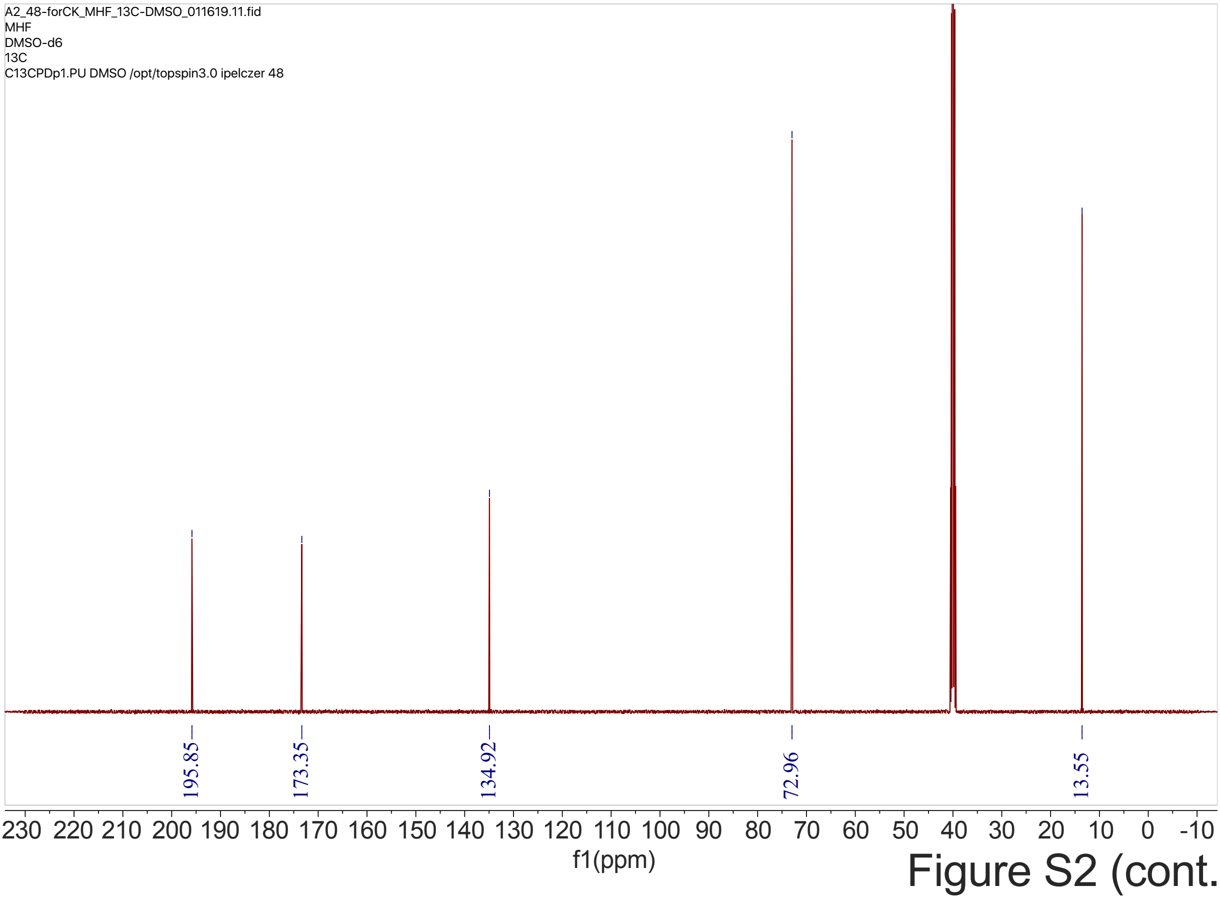

Figure S2 (cont.)
