# Supplementary material for: Saccharomyces cerevisiae Requires CFF1 To Produce 4-Hydroxy-5-Methylfuran-3(2H)-One, a Mimic of the Bacterial Quorum-Sensing Autoinducer AI-2
Source: mBio. 2021 Mar 9;12(2):e03303-20. doi: 10.1128/mBio.03303-20 (PMC8092285; doi:10.1128/mBio.03303-20)
Supplement: TABLE S1 [file mBio.03303-20-st001.docx]

**Table S1. Strains used in this study**

| *S. cerevisiae* strains | | |
| --- | --- | --- |
| **Strain Name** | **Genotype** | **Source** |
| MY8092 | WT *S. cerevisiae* | gift from Rose group |
| JSV1011 | MY8092 c*ff1*∆::KanMX | This work |
| JSV1131 | MY8092 *rps1b∆*::KanMX | This work |
| Y2 | wild strain isolated from rum | gift from Lewis group |
| Y12 | wild strain isolated from palm wine | gift from Lewis group |
| K11 | wild strain isolated from sake | gift from Lewis group |
| K1 | wild strain isolated from sake | gift from Lewis group |
| YJM440 | wild strain isolated from clinical isolate | gift from Lewis group |
| YJM308 | wild strain isolated from clinical isolate | gift from Lewis group |
| M32 | wild strain isolated from vineyard | gift from Lewis group |
| YPS1000 | wild strain isolated from oak | gift from Lewis group |
|  |  |  |
| *V. harveyi* strains | | |
| **Strain Name** | **Genotype** | **Source** |
| TL-25 | BB120 ∆*luxM*, ∆*luxPQ*, ∆*cqsS* | (29) |
| TL-26 | BB120 ∆*luxN*, ∆l*uxS*, ∆*cqsS* | (29) |
| *Z. rouxii* strains | |  |
| **Strain Name** | **Genotype** | **Source** |
| CBS 732 | WT | ATCC 2623 |
| JSV1250 | CBS732 *cff1∆*::KanMX | This work |
