# Supplementary material for: Saccharomyces cerevisiae Requires CFF1 To Produce 4-Hydroxy-5-Methylfuran-3(2H)-One, a Mimic of the Bacterial Quorum-Sensing Autoinducer AI-2
Source: mBio. 2021 Mar 9;12(2):e03303-20. doi: 10.1128/mBio.03303-20 (PMC8092285; doi:10.1128/mBio.03303-20)
Supplement: TABLE S2 [file mBio.03303-20-st002.docx]

**Table S2. Plasmids used in this study**

| **Plasmid Name** | **Plasmid Description** | **Source** |
| --- | --- | --- |
| pJSV789 | pRS416-p_CFF1_-HALO | This work |
| pJSV751 | pRS416- p_CFF1_-CFF1-HALO | This work |
| pJSV790 | pRS416- p_CFF1_-CFF1-E44A-HALO | This work |
| pJSV784 | pRS416- p_CFF1_-CFF1*_B. cinerea_*-HALO | This work |
| pJSV815 | pRS416- p_CFF1_-CFF1*_B. cinerea_*-E38A-HALO | This work |
| pJSV783 | pRS416- p_CFF1_-CFF1*_T. versicolor_*-HALO | This work |
| pJSV787 | pRS416- p_CFF1_-CFF1*_T. versicolor_*-E30A-HALO | This work |
| pJSV777 | pRS416- p_CFF1_-CFF1*_P. chlororaphis_*-HALO | This work |
| pJSV812 | pRS416- p_CFF1_- CFF*_U. tangerina_*-HALO | This work |
| pJSV809 | pRS416- p_CFF1_- CFF*_S. aureus_*-HALO | This work |
| pJSV806 | pRS416- p_CFF1_- CFF*_A. boritolerans_*-HALO | This work |
| pJSV810 | pRS416- p_CFF1_- CFF1*_A. fumigatus_*-HALO | This work |
| pJSV807 | pRS416- p_CFF1_- CFF1*_C. neoformans_*-HALO | This work |
| pJSV811 | pRS416- p_CFF1_- CFF1*_O. lucimarinus_*-HALO | This work |
| pJSV813 | pRS416- p_CFF1_- CFF1*_S. kowalevskii_*-HALO | This work |
| pJSV805 | pRS416- p_CFF1_- CFF1*_M. euhalobius_*-HALO | This work |
| pJSV808 | pRS416- p_CFF1_- CFF1*_A. hypogyna_*-HALO | This work |
| pJSV814 | pRS416- p_CFF1_- CFF1*_P. salinus_*-HALO | This work |
